# Recent Dominant Transposition Events Affect Gene Regulatory Regions, but Not Coding Sequences, in Polar and Brown Bear Genomes

**DOI:** 10.3390/cimb48060639

**Published:** 2026-06-20

**Authors:** Chris M. Njagi, James J. Kelley, Nikita Gulati, Naman S. Sijwali, Andrey Grigoriev

**Affiliations:** 1 Center for Computational and Integrative Biology, Rutgers University, Camden, NJ 08102, USA; cmn129@scarletmail.rutgers.edu (C.M.N.); jimkell@scarletmail.rutgers.edu (J.J.K.); nss235@scarletmail.rutgers.edu (N.S.S.); 2 Department of Biology, Rutgers University, Camden, NJ 08102, USA; ng884@scarletmail.rutgers.edu

**Keywords:** CAN SINE, regulatory evolution, structural variation, polar bear, brown bear

## Abstract

Transposable elements (TEs) are inserted into the genome and may change its properties; those occurring in or near regulatory regions may also alter gene expression. Given the challenges of detecting insertions in short-read sequencing, we analyzed structural variants in polar and brown bear genomes by a reciprocal alignment of one species’ sample genomes to a reference sequence of the other species, thus inferring TE insertion as the other genome’s “deletions”. With this approach, we detected short interspersed elements (SINEs) belonging to the CAN SINE family as dominant fixed TEs. We observed a non-random distribution of CAN SINE insertion positions near both protein- and RNA-coding genes, where TEs often overlap UTRs or occur in their vicinity. In contrast, SINEs avoid coding sequences, suggesting TE insertions that would disrupt such sequences are under purifying selection. We used black bear as an outgroup and determined that most of the CAN SINE insertions in the polar bear genome were derived, since they are not present in black or brown bear, while there is no dominant trend for CAN SINE insertions in brown bear relative to the outgroup. Many of the genes with UTRs affected by CAN SINEs are potentially relevant to the differences between the species (body shape, size, etc.) or to Arctic-adaptation phenotypes such as fur color, metabolism, and the immune system. This supports a model that CAN SINEs have contributed to regulatory evolution in bears and provides further evidence of such events across carnivore genomes in the animal kingdom.

## 1. Introduction

Transposable elements (TEs) are DNA sequences with the ability to change position in a genome [1]. These mobile DNA sequences proliferate through copy-and-paste or cut-and-paste mechanisms, accounting for a substantial fraction (often 35–50%) of mammalian genomes [2,3]. TEs are broadly grouped into class I retrotransposons, which transpose via an RNA intermediate (LINEs, SINEs, and LTR retrotransposons), and class II DNA transposons that move as DNA [4]. Although TEs were long regarded as genomic parasites or “junk DNA”, decades of work have shown that TE activity has shaped genome size, architecture, and the long-term accumulation of structural variation in vertebrates [5,6,7,8,9,10,11]. It is not confined to vertebrates, and we have shown the extent of such TE-driven variation across 3000 rice accessions [12].

Short interspersed elements (SINEs) are a type of TE, 80–400 bp long, and typically have three regions: a tRNA-related, tRNA-unrelated, and an A-rich region [13]. SINEs can be classified into several superfamilies. The CAN SINE family is broadly distributed across carnivores, but not in other mammals [13] and has contributed to shaping their genome evolution. SINEs are non-autonomous elements that depend entirely on the enzymatic machinery of Long Interspersed Nuclear Elements (LINEs) for their mobilization [14,15]. LINEs are autonomous retrotransposons, typically 6–7 kb in length, that encode ORF1p, an RNA-binding protein, and ORF2p, which provides both endonuclease and reverse transcriptase activities. During retrotransposition, a SINE RNA intermediate is reverse-transcribed and integrated into a new genomic location by the LINE-encoded machinery, generating hallmark structural signatures at the insertion site: target site duplications (TSDs) produced by staggered cleavage of the host DNA, and a 3′ poly(A) tail inherited from the polyadenylated RNA transcript [14,16]. This relationship directly links CAN SINE activity to the availability of functional LINE copies, yet most of the LINE TEs are likely degraded. Polar bear (PB) genome-wide surveys have identified ~1.2 million SINE insertions (8.4% of the genome) and nearly 1 million LINE remnants, 21.3% of the 2.3 Gb genome, of which a mere 535 LINE1 copies retained two intact open reading frames and appeared retrotransposition-competent [17]. We can estimate the level of LINE TE degradation from these numbers: given that LINE remnants account for ~493 Mb, the average remnant is ~0.5 kb, <10% of the intact 6–7 kb length, indicating extensive truncation.

Beyond their contribution to genome size and architecture, TEs are now recognized as a rich source of regulatory sequences. TE-derived promoters, enhancers, and transcription factor binding sites contribute a substantial fraction of regulatory elements annotated in the ENCODE datasets and can rewire gene regulatory networks [3]. Transposons inserted into or near a promoter region may alter gene expression patterns [18]. A particularly significant aspect of TE evolution involves structural modifications within regulatory regions, especially in 5′ untranslated regions (5′ UTRs), where insertions or deletions can alter transcription initiation, mRNA stability, and translational efficiency [19].

Structural variants (SVs) have likely contributed more to evolutionary adaptation and conservation than was previously appreciated, but their importance has often been underestimated because SVs have historically been more difficult to detect and interpret than SNPs [20,21]. As genome-scale and whole-genome data become increasingly available, conservation genomics can more directly assess adaptive potential, evaluate inbreeding and outbreeding risks, and identify fitness-relevant variation that SNP-only approaches may overlook [22,23,24,25]. This is especially important in closely related species or populations, where divergence is often concentrated in localized genomic islands of differentiation or haplotype blocks rather than distributed uniformly across the genome [21]. Because preserving evolutionary potential depends on identifying the variants that contribute to both present and future adaptation, integrating SVs into conservation analyses is increasingly recognized as essential [20,24]. In PBs, Arctic sea-ice loss and the potential for increased contact with brown bears (BBs) underscore the importance of defining lineage-specific adaptive variation, including SVs in regulatory regions [26,27]. Such information is needed both for predicting adaptive capacity and for assessing PB genetic rescue strategies that balance reduced inbreeding risk against maladaptive outbreeding or hybridization with BBs [28]. Genomes of the recently diverged PBs and BBs [17] allow for testing how recent SINE-associated SVs accumulate near regulatory regions during speciation, with potential effects on physiological and morphological phenotypes.

SVs corresponding to LINEs and SINEs were previously identified by aligning the assemblies of several dog breeds to the Greenland wolf [29]. CAN SINE insertion hotspots were found across canids using multiple sequence alignment and similarity to known SINEs [30]. Recent evidence includes temperature-dependent TE expression patterns that may be relevant to species experiencing different thermal environments [18]. These studies illustrate how TE-associated SVs can reveal the phylogenetic relationships behind the regulatory remodeling. However, such papers primarily compare arrays of TE events between species genomes and do not address the fine-scale distribution of TE-associated deletions relative to gene structure within a species.

By using whole-genome sequencing (WGS) data from PBs (*Ursus maritimus*) and BBs (*Ursus arctos*) and aligning the samples to reference sequences for both species, we can identify SVs and examine their distribution across bear samples to investigate population structure. Such SVs, overlapping known TEs, can identify specific recent transposition events directly, in contrast to standard probabilistic inferences based on the estimated age of TEs. In this study, we investigated the landscape of TE events in PB and BB genomes using a straightforward approach: detecting sequences present in one bear reference genome but absent in the other genome by aligning re-sequenced reads of each sample to a reciprocal reference. TE insertions are manifested as brown bear-specific deletions (BBSD) when reads are aligned to the PB reference and polar bear-specific deletions (PBSDs) when reads are aligned to the BB reference genome. Combined with the analysis of flanking sequences of these deletions, it allowed us to detect the exact coordinates of TEs and relate them to the nearest genes. Also, since our approach leverages read-level data from multiple individuals per species, we can distinguish homozygous from heterozygous SVs and identify those that are fixed among the sampled individuals of each species; we refer to these as fixed, while noting that sampling a limited number of individuals cannot establish fixation across the entire species.

Recent pangenome studies show that SVs can repeatedly underlie local adaptation, have measurable fitness consequences, and vary with demographic context and population size, reinforcing the importance of population-level sampling when interpreting structural divergence [31,32]. However, even with long-read, whole-genome alignment and pangenome approaches, assembly-based variant detection alone cannot precisely determine genotype frequencies or distinguish fixed divergence from segregating polymorphism without population-level read data [33,34]. Even when multiple assemblies are available, each represents a haplotype for that individual, and heterozygous variants or low-frequency alleles may be inconsistently represented or absent across assemblies [35]. A TE insertion present in 70% of assemblies from one lineage could represent either a derived insertion approaching fixation or an ancestral polymorphism, distinctions that require individual-level genotype calls from aligned reads. Our approach uses short-read population data to precisely genotype each variant in each individual, allowing us to identify candidate variants that are fixed (homozygous in 100% of sampled individuals) in one lineage and absent from the other, rather than inferring fixation from assembly presence/absence patterns alone.

Our approach reflects the fact that detection of insertions is a notoriously difficult problem for SV detection in short-read re-sequencing projects, while deletions have the highest reliability of detection [12]. Since we have reference sequences and samples of both PB and BB genomes, we circumvented the problem of insertion detection in our reciprocal analyses. We detected potential insertions into the PB genome as deletions in BB reads aligned to the PB reference and vice versa.

## 2. Materials and Methods

**Figure 1:**
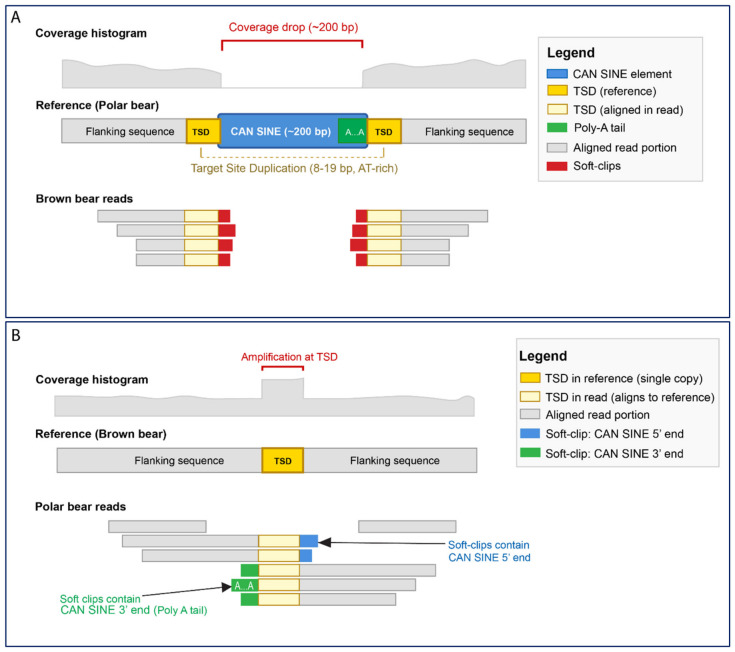
Detection and Validation of a CAN SINE Insertion. (**A**) Detection of a CAN SINE insertion in PBs as a deletion in BB reads aligned to the PB reference. (**Top**). The coverage histogram shows a characteristic drop across the CAN SINE region deletion. (**Middle**). The PB reference structure shows the complete CAN SINE element flanked by TSDs. TSDs are 8–19 bp AT-rich sequences that are duplicated during retrotransposition and serve as hallmark signatures of SINE insertion. The poly(A) tail at the 3′ end represents the characteristic adenine-rich terminus of SINEs. (**Bottom**). BB reads spanning the insertion breakpoints produce soft-clipped sequences (red) at the junction sites, where the reads extend into the CAN SINE but cannot be aligned to the BB genome. Split reads that span both breakpoints provide direct evidence of the deletion. (**B**) Reciprocal validation: detection of CAN SINE insertion in PBs by aligning PB reads to the BB reference. (**Top**). The coverage histogram shows an amplification peak at the insertion breakpoint. This amplification occurs because reads from both flanks of the CAN SINE element share microhomology with the single TSD sequence present in the reference. (**Middle**). The BB reference shows only a single copy of the TSD sequence at the insertion point. (**Bottom**). PB reads spanning the insertion boundary produce soft-clips containing the CAN SINE sequence. The 5′ junction shows soft-clips with the beginning of the CAN SINE element (blue), while reads from the 3′ junction show soft-clips with a poly(A) tail (green). Only the case where the CAN SINE is on the plus-strand version is shown. This case, as well as the case where the CAN SINE occurs on the minus strand, is shown in Supplementary Figure S3.

Complete command-line parameters for the software tools we used are available in the online GitHub materials (see README at https://github.com/grigoriev-group/genome-comparison (accessed on 15 June 2026)). WGS data for 10 BBs and 13 PBs [36], and 11 American black bears [37] were obtained from publicly available datasets (see Supplementary Table S1 for all accession numbers) and aligned using BWA-MEM [38] using default parameters to the current assemblies of PB and BB reference genomes (Supplementary Table S2) with a mapping quality threshold of greater than 20. SAM files from BWA alignment were converted to BAM, sorted, and indexed using SAMtools (v1.3.1) [39]. We employed a reciprocal analysis strategy of mapping genomes of one species’ samples to the reference of the other species to obtain a bidirectional view of lineage-specific structural variation (Figure 1). We used GROM, a variant caller that integrates multiple lines of evidence (discordant read pairs, split reads, soft-clipped reads, and read depth), to generate SV calls for each sample [40]. We identified deletions with high-quality scores and lengths of 50 bp to 10,000 bp for the general overview of the deletion SV landscape, narrowed down to those within 5000 bp of annotated exons for a refined analysis of regulatory regions, and used deletions within 1000 bp to assess their enrichment around gene features.

To consolidate redundant calls representing the same underlying deletion events and to identify deletions shared across multiple individuals, we performed density-based clustering using a modified DBSCAN (Density-Based Spatial Clustering of Applications with Noise) algorithm [41,42]. Deletions from all samples within each species were merged into unified datasets, and clustering was performed based on genomic proximity and size similarity. Two deletions were considered part of the same cluster if their start and end positions differed by ≤50 bp, reflecting typical uncertainty in breakpoint detection from short-read sequencing data. We also required that clusters should not overlap by more than 30% to be considered unique. To obtain reliable deletion SV calls, we required at least half of the deletions in each cluster to have the highest quality score (9999 in GROM output) and for the coefficient of variation in deletion sizes to be <0.2. Variants were classified as species-specific if present in ≥50% of one species’ genomes and absent from all genome samples of the other species. This read-level resolution also lets us examine the allele frequency spectrum of these variants across individuals: a permissive prescreen (a deletion segregating in roughly half of one species’ samples) admits polymorphic candidates, whereas the fixed deletions set was restricted to those present in 100% of one species’ samples in the homozygous state and absent in the other species’ samples.

We then generated precise borders of the identified SVs using the split-read approach of Pindel [43] using default parameters. For a follow-up validation of algorithmic prediction and comparison of GROM and Pindel results, we used the following manual curation method for the deletions obtained after filtering. Each candidate event was evaluated on: (i) a read-depth decrease across the deletion interval with retained events showing interior coverage ≤ 15% of the mean flanking depth; (ii) split-read or soft-clipped-read clustering at the breakpoints with ≥4 such reads summed across the two junctions (within ±20 bp of the inferred breakpoints); (iii) callable coverage with mean depth ≥ 5× on each flank at MAPQ ≥ 20, distinguishing a true absence from a low-coverage no-call; and (iv) reciprocal-reference support (a corresponding insertion signature when the other species’ reads were mapped to the assembly). A sample was scored as carrying the deletion only when the depth and split-read criteria were jointly satisfied. For every locus we report, per sample, the depth across the deletion and its flanks, the depth-drop ratio, split/soft-clipped read support at each breakpoint, a callable-status flag, and the resulting call (Supplementary Table S5 available at https://github.com/grigoriev-group/genome-comparison (accessed on 15 June 2026)). A locus was retained as species-specific only where the deletion was fixed across all samples of the focal species and absent from every sample of the reference species. We used Genome Navigator (an in-house SV visualization tool, illustrated in Supplementary Figure S1) and followed the principles shown in Figure 1 to determine if these deletions were homozygous and to confirm consistent breakpoint evidence across samples. Events with an empty interior (no reads aligned within the deletion) without sufficient breakpoint support were resolved this way. The coordinates of deletion breakpoints were then used to extract ±200 bp flanking sequences from the reference genome and used to query the reciprocal bear reference with BLASTn [44]; concordant left/right flank placements in the expected orientation and absence of the intervening reference sequence supported the deletion call.

To determine the ancestral versus derived state of species-specific CAN SINE insertions, we used the American black bear as an outgroup. For each species-specific SV identified in PB or BBs, we determined whether an orthologous SV was present in outgroup samples. Deletions present in the outgroup were interpreted as representing the ancestral state, indicating that the corresponding sequence represents a derived insertion in the focal lineage.

Gene associations were determined by comparing SVs with respective bear reference gene models using BEDTools v2.30.0 [45]. We identified overlapping genes where deletions intersect gene boundaries and nearby genes within 5000 bp of the nearest gene boundary, upstream of the TSS and downstream of the TES, using NCBI RefSeq GFF annotation [46]. CAN SINE sequences were identified by SINEBase alignment [47]. Species-specific CAN SINE deletions were defined as those present in ≥50% of samples from one species and absent from all samples of the other species. Reference sequences of (20 bp) flanking each deletion were extracted, and TSDs in each case were identified as the longest *k*-tuple where the last *k* bases of the left flank of a deletion exactly matched the first *k* bases of its right flank. We required potential TSDs to be 8–19 bp in length for consistency with CAN SINE insertion signatures. For each deletion, we cataloged deletion coordinates, deletion size, sample support, and gene associations for both species. Functional annotation utilized the Mouse Genome Informatics (MGI) database phenotype associations [48], GeneCards [49], the Rat Genome Database (RGD) [50], and MalaCards human disease databases [51]. Mice and humans are both very distant from bears, but these databases do point to pathways that are potentially impacted by mutations of affected genes.

## 3. Results

This section is organized as follows. We start by summarizing the composition and size distribution of the variants by deletion size, highlighting a dominant peak around the characteristic mean length for SINEs and a secondary peak for LINEs. We then focus on a curated set of ~200 bp CAN SINE–associated events that intersect exons and regulatory regions, describing representative cases with plausible functional impact. Next, we evaluate how TE-associated variants are distributed across gene features (untranslated and regulatory regions), present the sets of species-specific deletions SVs identified using the reciprocal reference genome alignment, and finally assess functional enrichment of affected genes to identify biological pathways and phenotypic themes potentially relevant to PB-BB divergence.

### 3.1. Overview of Species-Specific Deletions Identifies CAN SINE as the Dominant TE Events

**Figure 2:**
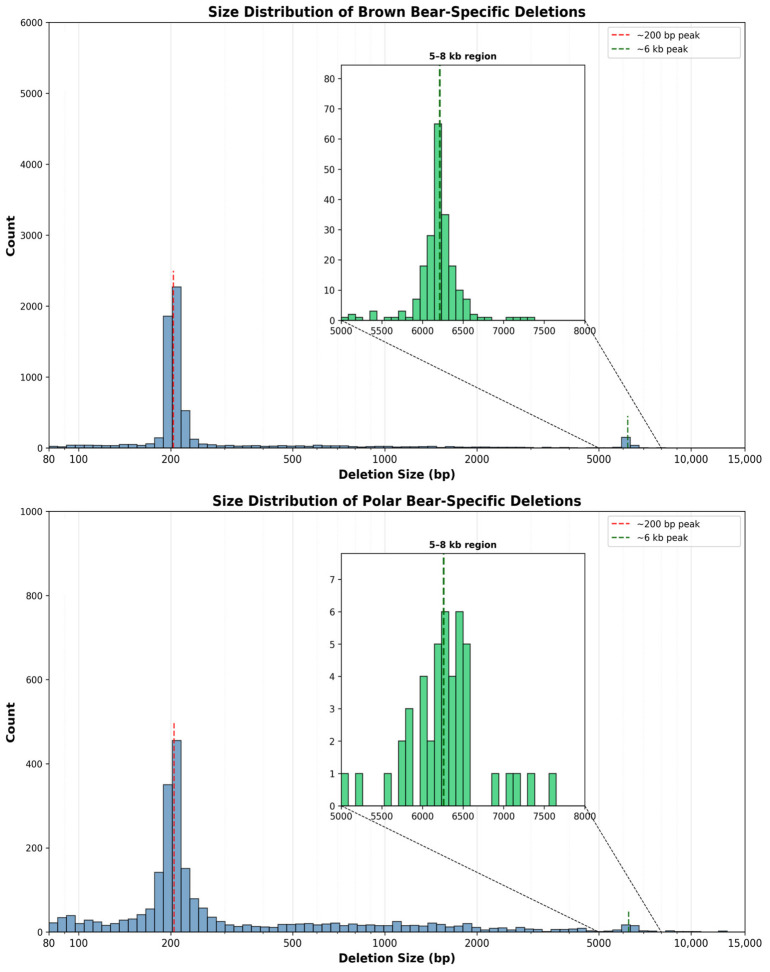
Deletions size distribution. (**Top**). Size distribution of BBSDs (*n* = 6677) plotted on a logarithmic scale. (**Bottom**). Size distribution of PBSDs (*n* = 2496). In both plots, shorter deletions are strongly predominant, with a median of ~200 bp consistent with CAN SINEs’ length (red dashed line). A distant secondary peak is visible at ~6250 bp (green dashed line), consistent with full LINE lengths (also shown as an inset).

We extracted species-specific deletions from the WGS by aligning BB samples to the PB reference genome and vice versa, identifying SVs (see Section 2) with our GROM algorithm [40] and focusing on deletions present in 100% of samples of one species and absent in the other. We identified 6677 BBSDs when comparing the BB samples to the PB reference genome and 2496 PBSDs when comparing the PB samples to the BB reference genome. For each, we report the supporting breakpoint evidence (soft-clipped-read support and breakpoint confidence) and start/end read-depth ratios (Supplementary Datasets S1 and S2). We observed dominant deletions ~200 bp in length (Figure 2) in both bear species, consistent with the typical length of CAN SINEs. A secondary peak at approximately 6250 bp is consistent with the length of intact LINEs, at substantially lower frequency than the CAN SINE class. The ~200 bp deletion sequences matched CAN SINEs or their reverse complement by alignment to the CAN SINE consensus in SINEBase [47], identifying them as the dominant TE events in bear genomes. All deletion sequences carried a 3′ poly(A) or a 5′ poly(T) tail, a hallmark feature of SINEs.

In the sections below, we often refer to TE events as SVs of type “deletions” and to fixed TEs as “fixed deletions”, since this type is what we mainly observe when comparing aligned samples to a reciprocal reference using variant callers. Deletions in the genome of a sample may be indicators of insertions in the reference genome. However, detection of insertions is much harder, as mentioned above, complicating the determination of whether they are fixed. For their actual classification as deletions or insertions, see the section on Section 3.8.

### 3.2. CAN SINE SVs Overlapping Exons

Nineteen TEs overlap exons or UTRs (Table 1) and are likely fixed (being absent in all BB samples aligned to the PB reference), indicating strong lineage-specific differentiation. These genes are significantly enriched for adaptation phenotypes distinguishing PBs from BBs, such as body weight (*BBS10*, *PDSS2*), metabolism (*RNF213*, *PIGH*, *MDH2*), and immunity (*ZBTB37*, *SMARCA4*). There was also a deletion in one exon of a non-coding transcript variant X3 of the *PDIK1L* gene (misc_RNA XR_569523.2, not shown in Table 1 as the deletion is intronic for two protein-coding transcript isoforms). Interestingly, the *PDIK1L* gene is relevant for hair pigmentation.

In PB samples aligned to a BB reference genome, we identified seven fixed CAN SINE deletions overlapping exons with UTR regions (Table 2). Several of the affected genes have Mouse Genome Informatics (MGI) phenotype associations [48] potentially relevant to Arctic adaptation. The concentration of SVs in genes affecting metabolism (*RHBDD1*, *UACA* for cholesterol and *GPN2* for carbohydrate metabolism) parallels the functional enrichment observed among the BBSDs above, with phenotypic categories that distinguish the two bear species.

### 3.3. CAN SINE Deletions Upstream of 5′ UTR

In the BB genome, 20 SVs are fixed and reside within 5000 bp upstream of the transcription start site (TSS), a window that typically encompasses core promoter elements, proximal enhancers, and transcription factor binding sites (Table 3). Several genes with deletions in potential promoter regions are known to affect coat pigmentation or skin (*CHIC2*, *DLGAP1*), craniofacial/skeletal traits (RANBP3L), or limb morphology (*RECK*, *SPATA6*). Interestingly, *EFHC2* and *OCRL* are on the X chromosome and are therefore hemizygous in males, meaning any regulatory changes caused by these upstream deletions would be immediately exposed to selection rather than masked by a second allele.

In the PB genome, 14 deletions are fixed and are all located within 5000 bp upstream of TSS (Table 4). Several affected genes have functions relevant to arctic survival, including body composition and fat storage (*GALNT18*, *MTLN*), lipid and cholesterol metabolism (*KLRD1*, *SULT2B1*, *MTLN*), carbohydrate metabolism and insulin signaling (*SLC25A51*, *SERTAD1*), thermal pain nociception (*MMP24*), and immunity (*CUNH15orf39*). *CT83* is also on the X chromosome.

### 3.4. CAN SINE Deletions Downstream of 3′ UTR

We also considered TE events downstream of the transcription end site (TES). Insertions in this region may carry additional 3′ regulatory elements, including polyadenylation signals and binding sites for RNA-binding proteins or small RNA. We identified 14 fixed BBSDs (Table 5) and 7 fixed PBSDs downstream of 3′ UTRs (Table 6). Many of these deletions affect genes with phenotypes potentially relevant to the divergence of the two species. There are genes influencing bone development (*EPG5*, *GLI2*, *IHO1*), immune function (*EPG5*, *HAUS1*, *MAP4K3*, *TAF7*, *SEMA4A*), lipid metabolism (*HAUS1*, *FBRS*), digestive system development (*IHO1*), and hair pigmentation (*FAM24B*, *ZNF706*). A tRNA gene, *TRNAD-GUC*, aspartic acid (GUC anticodon) is seen in Table 5, but its significance here is unclear.

### 3.5. TEs and Gene Features

**Figure 3:**
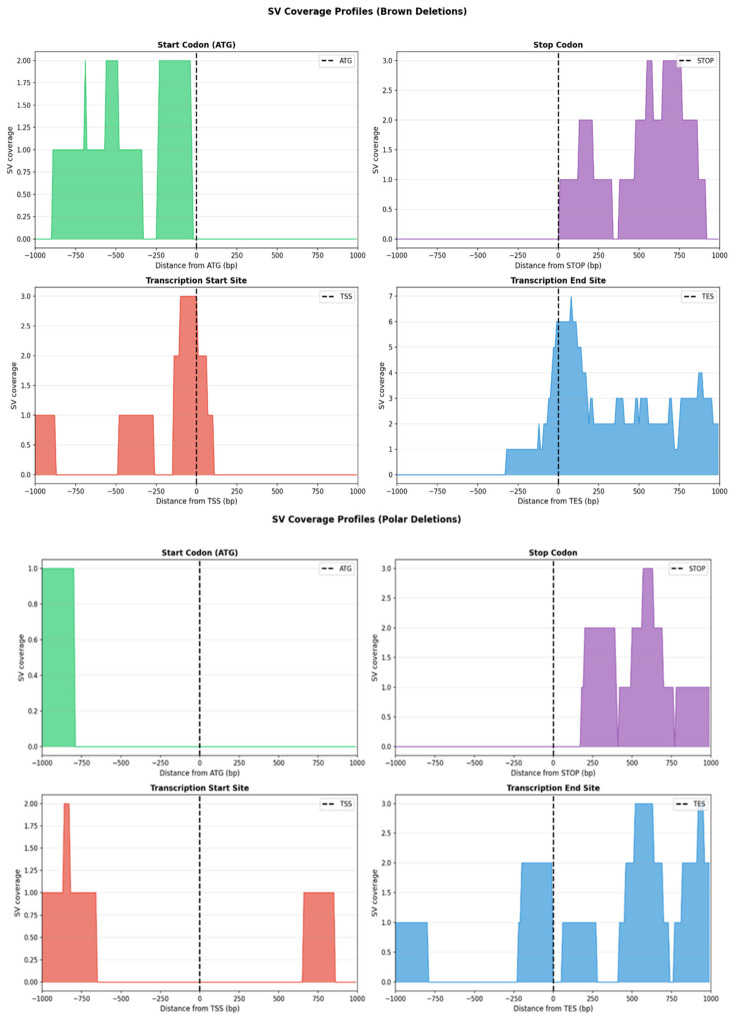
Species-specific deletion profiles relative to gene features. Deletion density is plotted as a function of distance from four gene features: (TSS), (TES), start codons (ATG), and stop codons. (**Top Panel**). Deletion profiles of BBSDs aligned to the PB reference genome. (**Bottom panel**). Deletion profiles of PBSDs aligned to the BB reference genome. Direct CDS-overlap testing (using average GROM coordinates): 2 of 3400 brown-specific and 1 of 865 polar-specific SINE events overlapped a CDS, versus 214.0 ± 14.3 and 50.8 ± 6.9 expected under a gene-proximal permutation null (10,000 permutations), a ~110-fold and ~50-fold depletion (empirical *p* < 1 × 10^−4^ in both directions). On refined breakpoint inspection in Genome Navigator, none of the three apparent intersects a CDS.

To assess the spatial relationship between TE-associated events and gene features, we analyzed their distribution relative to TSS, TES, start codons (ATG), and stop codons (STOP). We observed a striking pattern of TE avoidance near CDS boundaries: none of the 81 events crossed CDS borders demarcated by ATG or STOP (Figure 3). This pattern was very significant, e.g., for BBSDs, *p* < 0.026 near ATG and *p* < 0.009 near STOP (chi-squared test). Direct intersection of all SINE-associated candidate events with annotated CDS confirmed strong CDS depletion. BEDTools identified only 2 of 3400 brown-specific events (polar reference) and 1 of 865 polar-specific events (brown reference) overlapping CDS, versus the respective 214.0 ± 14.3 and 50.8 ± 6.9 expected under a gene-proximal permutation null (10,000 permutations; empirical *p* < 1 × 10^−4^ in both directions). However, all three apparent overlaps are ≤12 bp terminal grazes of a CDS boundary, and on inspection in Genome Navigator, none of them intersect CDS; hence, no SINE-associated event falls within the coding sequence. This pattern suggests that TEs disrupting CDS are under purifying selection. In contrast, there was a more uniform distribution (e.g., for BBSDs *p* = 0.17, chi-squared test) around transcriptional boundaries (TSS and TES), with a substantial “spillover” into flanking regulatory regions. This broader distribution around TSS/TES is consistent with TEs targeting promoters, enhancers, and UTRs, where TEs may modulate gene expression without abolishing gene function. The concentration of TEs in these regions supports a model of CAN SINEs contributing to species-specific regulatory evolution.

### 3.6. CAN SINE Flanking Sequence Analysis

Having established that the deletions match known CAN SINEs and that all elements had a poly(A/T) tail, we examined the left and right flanks of all entries in Table 1, Table 2, Table 3, Table 4, Table 5 and Table 6 for two hallmarks of retrotransposition: TSD repeats on both ends, usually of length 8–20 bp [52,53,54], and an A/T-rich insertion-site signature. The flanking sequences revealed strong signatures of CAN SINE retrotransposition in both species. In total, 100% of BBSDs had TSDs of mean length 15 bp and >64% AT content. Among 28 PBSDs, 26 have TSDs of mean length 16 bp and >67% AT content. This is consistent with the AT-rich insertion site preference of SINEs (much higher than the GC of 41–43% in bear genomes overall). The PBSD sites largely contain sequences similar to the TSD in the BB genome reference and vice versa (Supplementary Table S3). To estimate the chance of the false-positive rate under AT-rich composition, each locus’s flanks were shuffled 10,000 times, preserving local mononucleotide composition, and the anchored match was recomputed, giving a per-locus chance probability. In total, 80 of the 81 TSDs are unlikely to arise by chance (*p* < 0.05; 76 at the 10,000-permutation floor, *p* < 1 × 10^−4^); the single exception is the one locus with no detectable TSD. These probabilities are reported in Supplementary Table S3.

**Figure 4:**
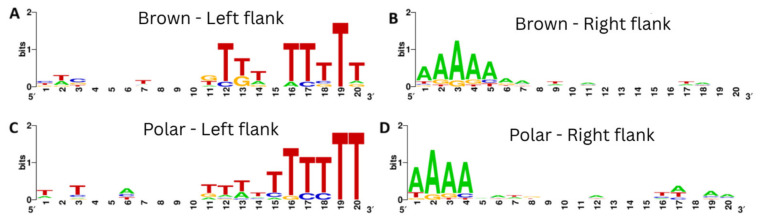
Sequence logos of 20 bp breakpoint-flanking reference sequence for loci with A/T-rich insertion-site signatures. (**A**,**C**) A total of 20 bp immediately upstream of the breakpoint (left flanks), illustrating complementary T—enrichment near the junction. (**B**,**D**) A total of 20 bp immediately downstream of the deletion breakpoint (right flanks), illustrating A—enrichment near the junction. Panels (**A**,**B**) correspond to brown deletions mapped to the polar reference; panels (**C**,**D**) correspond to polar deletions mapped to the brown reference. Letter heights (bits) reflect positional nucleotide conservation. Logos were generated on weblogo.berkeley.edu (Last accessed on 8 March 2026).

We then examined the breakpoint-flanking composition for the A/T-rich signature expected at CAN SINE target sites (Figure 1 and Figure 4). In total, 50% of PBSDs show T-runs at the left flank (≥4 Ts in the last 6 bp), and 50% have A-runs at the right flank start (≥4 As in the first 6 bp), with both runs in four deletion sites (near *EPN2*, *SULT2B1*, *ZNF706*, *TAF7*); overall, 24 of 28 (85.7%) show runs on at least one flank. Among BBSDs, 26 (49.1%) have T-runs on the left, 15 (28.3%) have A-runs on the right; *SEMA4A* and *ZSCAN2* show both runs; overall, 39 of 53 (73.6%) sites have runs on at least one flank.

Estimating the per-flank single-nucleotide background directly from the flanking sequences (T ≈ 0.34 in PB and 0.37 in BB left flanks; A ≈ 0.35 in PB and 0.30 in BB right flanks), the expected probability of a ≥4-nt run on at least one flank is ~0.21 (PBSD) and ~0.20 (BBSD). The observed rates—24/28 (85.7%) for PBSD and 39/53 (73.6%) for BBSD—correspond to 4.0- and 3.8-fold enrichment, with one-sided binomial locus-level *p* = 6.3 × 10^−13^ (PBSD) and 2.6 × 10^−17^ (BBSD). Thus, the enrichment reflects the A/T-rich insertion-site signature (target-site composition, together with the SINE poly(A) terminus) rather than genome-wide background AT content. The remaining loci that did not meet the ≥4 threshold nonetheless carry other CAN SINE hallmarks, including TSDs within the expected size range and, most importantly, deleted sequence identity to known CAN SINEs. Together, our observations confirm that deletions in Table 1, Table 2, Table 3, Table 4, Table 5 and Table 6 represent CAN SINE retrotransposition footprints; insertions in one lineage that are absent in the other.

### 3.7. Reciprocal Validation of Insertion-Deletion Events

We then checked if observed deletions may represent insertion events in the reciprocal lineage. GROM detected 80 out of 81 (98.8%) deletions in Table 1, Table 2, Table 3, Table 4, Table 5 and Table 6 as insertions in one or more samples. Typically, insertion calls were successful in samples with the highest sequencing quality, although the variable number of such supporting samples did not allow us to confirm fixed insertions based solely on GROM calls. This is consistent with the recognized difficulty of insertion detection by current short-read variant callers [12].

We visually inspected each of the cases in Table 1, Table 2, Table 3, Table 4, Table 5 and Table 6 and observed that for 12 genes, exon positions and isoform annotations differ significantly in the two species, so we could not rely on gene names alone for our comparison. To enable systematic analysis, we utilized manual curation (see Section 2 and Figure 1) to identify regions that potentially contain insertions in that reciprocal lineage.

Among the 53 BBSDs (Table 1, Table 3 and Table 5), this approach identified 52 corresponding insertions when PB genome samples were mapped to the BB reference. The single exception, *PARVB*, is a likely artifact of assembly or incomplete annotation as discussed below. The insertions exhibited characteristic CAN SINE signatures with A- or T-rich termini at the predicted coordinates, soft-clipped reads at insertion boundaries, and coverage amplification from reads aligning to both flanks of the TE. Among the PBSDs (Table 2, Table 4 and Table 6), we identified corresponding insertions in the BB reference for all 28 loci (100%). These findings are presented in Supplementary Table S4.

**Figure 5:**
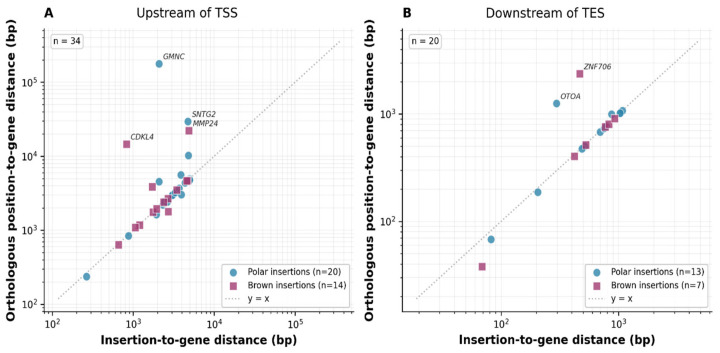
Conservation of CAN SINE insertion positions relative to genes. Scatter plots comparing the distance from species-specific CAN SINE insertions to the nearest gene in the source genome (x-axis) versus the distance from the orthologous position to the same gene in the genome lacking the insertion (y-axis). (**A**) Upstream of the TSS. (**B**) Downstream of the TES. Blue circles: PB-specific insertions. Magenta squares: BB-specific insertions. Dotted gray line: identity (y = x). Reciprocal validation recovered the corresponding insertion for 52 of 53 BBSD-associated events and 28 of 28 PBSD-associated events (80/81; 98.8%). The single exception (PARVB) and the positional outliers off the diagonal likely reflect annotation or assembly differences between the two reference genomes rather than failed validation.

For most deletions, the insertion positions in the reciprocal bear reference correspond to the expected genomic regions relative to gene features, with distances to the nearest UTR or gene boundary consistent with the deletion positions in the other bear reference (Figure 5, Supplementary Table S4). However, 12 outliers deviate substantially from the expected positions (particularly in the upstream regions), as mentioned above. We identified distinct patterns that explain the positional offsets (detailed in Supplementary Table S4 and Supplementary Figure S2, “Positional Discrepancy Cases”). For example, *GMNC* shows an extreme offset (~177 kb) reflecting differences in annotated gene size. *DLGAP1* displays a ~10 kb offset while *MMP24* exhibits a ~17 kb discrepancy reflecting incomplete gene models in the BB reference assembly. The insertion for *SNTG2* was found to be upstream of the 5′ UTR in one isoform and downstream of the 5′ UTR in an intron of another isoform. These substantial differences likely reflect a combination of assembly quality issues, incomplete gene models, and genuine structural variation between lineages. Additional outliers arise from possible mis-annotations, such as UTR length differences (>1 kb for some 3′ UTRs) or multiple isoforms found in one assembly but not the other. In the case of *ZSCAN2*, the 5′ UTR is discontinuous, and the SV is located between the two portions of the 5′ UTR (Case 5). For four loci (*RHBDD1*, *UACA*, *RNF170*, and *SNTG2*), the insertions fall upstream of the 5′ UTR in one isoform and downstream of the 5′ UTR in an intron of another isoform, reflecting differences in isoform-specific UTR boundaries (Case 7 in Supplementary Table S4 and Supplementary Figure S2).

For *PARVB*, BLASTn failed to identify a corresponding insertion in the BB reference genome. We also noted that *PARVB* is absent from the current BB annotation, despite five distinct isoforms found in PB. A high degree of conservation at the parvin C-terminus across PBs, humans, and mice strongly argues against functional gene loss. This likely reflects an incomplete annotation in the BB reference rather than true gene absence, as the parvin family shows a complex genomic architecture with non-canonical polyadenylation signals and variable 3′ UTR isoforms that confound automated annotation pipelines [55].

### 3.8. Outgroup Analysis for Ancestral State Inference

To determine the ancestral state of the reported TE events, we conducted an outgroup analysis using American black bear samples. Among the 52 PB-specific insertions, 48 loci (92.3%) were confirmed absent from all the outgroup samples, confirming that these insertions represent derived states in the PB lineage. Four loci (*RECQL4*, *ZSCAN9*, *RGS22*, and *SPATA6*) showed the CAN SINE insertion present in the outgroup. These cases likely represent incomplete lineage sorting or secondary loss in BBs.

In total, 20 out of 28 insertions (71.4%) were deemed derived in BB (but only two out of the seven that overlapped exons), as they had no orthologous insertions in any outgroup sample. Two additional loci showed TE polymorphism: a CAN SINE insertion in some of the outgroup samples and a corresponding deletion in others. *CFLAR* had five outgroup samples with a deletion, two with an insertion, and four with heterozygous insertions or deletions (see Figure S4 for a visualization of heterozygous calls in reciprocal alignments). For *CLDN17*, two outgroup samples had a deletion, and the remaining nine had an insertion. Six loci (*GPN2*, *KLRD1*, *USB1*, *SULT2B1*, *ORC6*, and *RHBDD1*) showed an insertion present in the outgroup, suggesting these may represent ancestral insertions shared across bear lineages or incomplete lineage sorting.

The outgroup analysis reveals that the majority of species-specific CAN SINE insertions in both PB and BB represent derived gains rather than ancestral states. This pattern suggests that active CAN SINE retrotransposition occurred in both lineages following their divergence, with the PB lineage accumulating more derived insertions, potentially associated with adaptation to Arctic environments.

## 4. Discussion

Our comprehensive analysis of SVs in BBs and PBs reveals a likely contribution of CAN SINE-associated insertions to genomic divergence between these species. CAN SINEs appear to have played a major role in PB adaptation to the Arctic environment based on the number of genes affected by fixed insertions, and functions of many of these genes are related to the phenotypic differences between the two species. This study finds that PBs accumulated substantially more derived CAN SINE insertions (47 of 52 loci, 90.38%) than BBs (20 of 28, 71.4%), suggesting more active retrotransposition in the PB lineage following divergence.

We detected 6677 BBSD calls and 2496 PBSD calls, a 2.7-fold difference, which underscores the variance in the SV landscape among these two bear populations. This asymmetry reflects the interaction between reference-dependent ascertainment and the distinct demographic histories of these lineages [28,36]. Because a “deletion” is defined purely relative to the reference assembly, the direction of asymmetry reveals which lineage accumulated more fixed sequence gains versus losses. We observed substantially more BBSD calls, most of them (47/52, 90.38%) being recent insertions in PB based on outgroup analysis as opposed to BB insertions (20/28, 71.4%). Bias on such a scale has already been reported for SNPs (but in the opposite direction): reflecting their approximately 3-fold lower genetic diversity, PBs harbor only 2.6 million private SNPs (variants unique to one species and absent in the other) compared to 7.7 million in BBs [36]. Given such low genetic diversity as a consequence of population bottlenecks [28,56], PBs would also be expected to exhibit lower heterozygosity of TE insertions.

The severe population bottleneck and persistently low effective population size (N_e_) in PBs [28,36] have important implications for the fixation dynamics of TEs. Small N_e_ is typically associated with the fixation of mildly deleterious mutations and the loss of rare beneficial variants through drift [57]. However, the supply of potentially adaptive variation in small populations is constrained by low per-nucleotide mutation rates, the small proportion of mutations that are beneficial, and the limited number of individuals generating new mutations [57,58]. TEs have been proposed as a mechanism to resolve this constraint: by generating structural mutations at rates that may exceed nucleotide substitution rates, they could provide a disproportionately rich source of beneficial variation in bottlenecked populations [59]. Indeed, recent evidence demonstrates that TEs can maintain genome-wide heterozygosity and generate adaptive variation even under intense inbreeding [60]. Each CAN SINE insertion introduces ~200 bp of novel regulatory sequence in a single event, a mutational input qualitatively different from point mutations [61]. This framework suggests that CAN SINE retrotransposition could have been especially impactful during PB adaptation, compensating for the reduced supply of adaptive point mutations with a higher per-event rate of regulatory innovation.

The predominance of ~200 bp deletions in the two genomes is consistent with the known characteristic length of CAN SINEs in carnivores [13]. While the ~6000 bp peak in Figure 2 is consistent with nearly intact LINEs (and populated areas between 200 and 6000 bp may contain very numerous LINE remnants), detailed analysis of these larger events is beyond the scope of this study. The LINE remnants may host multiple nested TE insertions or deletions that removed partial LINE sequences along with flanking genomic DNA, a pattern commonly observed in TE-rich genomic regions [62]. The lower abundance of full-size LINE TEs may also reflect purifying selection against large SVs that disrupt functional sequence, reduced detection power among repetitive regions, or greater heterozygosity of LINE insertions with fewer events reaching our fixation threshold.

SINEs have been reported to be enriched (and LINEs depleted) in transcript regions [18], a pattern compatible with our observations. The presence of insertions in UTRs and the absence of insertions in protein-coding exons are consistent with purifying selection against disruptions to the CDS [63]. CAN SINE insertions in both UTRs and upstream regions suggest influence on gene regulation through multiple mechanisms, possibly affecting mRNA stability, localization, and translational efficiency or altering transcription factor binding sites and enhancer elements.

The observed 28 PB and 17 BB CAN SINE fixed insertions within 5000 bp of gene 5′ UTRs may directly impact transcriptional regulation. TE insertions can alter gene expression by introducing or removing transcription factor binding sites, changing chromatin accessibility, or providing alternative TSSs. Indeed, several genome-wide studies show that polymorphic TE insertions can be expression quantitative trait loci or alternative promoters that drive tissue- or tumor-specific transcription [64,65]. Further, 14 PB-specific and 7 BB-specific fixed insertions are downstream of 3′ UTRs, a position where longer UTR isoforms may carry additional polyadenylation signals and binding sites for RNA-binding proteins or microRNAs.

SINEs have been reported to show orientation bias relative to host gene transcription, with antisense insertions tolerated more readily in regulatory regions [66]. Though our sample size is limited, we tested whether CAN SINEs in bear genomes show a similar pattern. Neither species showed any significant deviation from random orientation. Sense orientation here refers to the transcription direction of the TE being the same as that of the host gene and vice versa. In BB, 56.6% (30/53) were in antisense orientation, while in PB, 60.7% (17/28) were in antisense orientation. However, a regional pattern emerged in PB: UTR-overlapping insertions: 5′ UTR-associated CAN SINEs showed 66.7% (6/9) antisense orientation, whereas 3′ UTR-associated elements showed only 33.3% (4/12) antisense orientation. PB-specific insertions showed substantial strand asymmetry in gene direction: UTR-overlapping insertions significantly favored genes on the minus strand (14 vs. 5, *p* < 0.039, chi-squared test), while upstream deletions slightly favored genes on the plus strand (13 vs. 7, *p* < 0.17, chi-squared test). In contrast, BB-specific insertions showed no such bias in gene strand direction in either category.

Cross-referencing affected genes from Table 1, Table 2, Table 3, Table 4, Table 5 and Table 6 with mammalian phenotype annotations from the MGI database [48], GeneCards [49], Rat Genome Database [50] and disease associations from MalaCards [51] showed that the functional profile of affected genes converged: (i) body size, skeletal proportions, and craniofacial morphology; (ii) energy metabolism, lipid handling, and thermoregulation; (iii) integumentary traits including coat color and hair structure; (iv) body immunity [36,67]. These categories align with the known morphological and physiological differences between PBs and BBs, so it is tempting to speculate that CAN SINE insertions contributed to adaptive phenotypic changes during Arctic colonization.

Several genes show particularly strong associations with Arctic-relevant phenotypes, such as hyperpigmentation (*SLC29A3*) [68]. *BBS10* regulates leptin levels and energy homeostasis [69]. Given that PBs show reduced hibernation compared to BBs, alterations in leptin signaling could contribute to their divergent metabolic strategies. *CHIC2* is associated with abnormal coat/hair, linking it to the dramatic coat color differences between the two species. Mice engineered with a targeted mutation in the *GALNT18* gene associated with a PB-specific upstream insertion demonstrated increased lean body mass and decreased total body fat compared to wild-type mice (MGI). We also observed a TE event near *MTLN*, which encodes mitoregulin, a small mitochondrial microprotein involved in regulating mitochondrial membrane potential, fatty acid beta-oxidation, and cardiac muscle morphology. Notably, several SVs affect genes on the X chromosome (*EFHC2* and *OCRL* in BBs; *CT83* in PBs), where hemizygosity in males ensures that any regulatory changes are immediately exposed to selection rather than masked by a second allele, potentially accelerating adaptive evolution. Overall, SVs that may affect metabolic and morphological pathways occurred in both lineages, likely reflecting adaptation to divergent ecological niches: Arctic marine carnivorous mode for PBs versus temperate terrestrial omnivorous mode for BBs.

A limitation of this study is that the quality of the analyzed samples may affect our results; it is difficult to establish a fixed SV in a poorly represented genome region. Importantly, for the SVs we detected, the reciprocal analysis allows the direct identification of species-specific structural variation and demonstrates that both species have accumulated distinct TE-associated changes since their divergence was less than 500,000 years ago [36].

Outgroup analysis using American black bear samples confirms that the vast majority of observed species-specific events represent derived insertions, especially in PBs, rather than ancestral losses, consistent with ongoing CAN SINE retrotransposition. A few insertions were shared between the focal species and the outgroup, while absent from the sister species. This pattern is compatible with incomplete lineage sorting during the rapid radiation of these bears [56] or secondary loss in the sister lineage. Notably, among the seven BB-specific insertions overlapping UTRs (Table 2), only two were derived, while four were shared with the outgroup, and one showed a mixed ancestral state. This indicates a loss in the PB lineage, affecting genes associated with metabolism and body size (e.g., *GPN2*, *ORC6*, *RHBDD1*). Independent parallel insertion is unlikely given the sequence specificity of retrotransposition. The reciprocal comparison also exposed discordant gene models between the two reference assemblies, most strikingly the complete absence of *PARVB* from the BB annotation despite high protein sequence conservation of the *PARVB* PB locus in animal genomes. Such annotation asymmetries complicate positional comparisons and underscore the need for improved reference completeness in non-model species.

## 5. Conclusions

Here, we utilized available short-read WGS data, combined with the updated bear reference genomes, to provide a robust reciprocal cross-species alignment framework for detecting species-specific SVs and identified fixed derived TE insertions potentially affecting the regulation of 67 genes. These SVs are preferentially located in and around gene-regulatory regions and depleted from coding sequences, with the majority polarized as derived in the polar lineage by outgroup comparison. Together, these patterns support a contribution of recent CAN SINE retrotransposition to regulatory divergence between the two species. The loci identified here, particularly insertions affecting the UTRs of genes linked to metabolism, pigmentation, and immunity, provide concrete candidates for functional follow-up. The reciprocal-reference strategy can be transferred readily to other recently diverged taxa. Future work should include expanded sample sizes from additional populations, functional validation of regulatory effects through gene expression analysis, and an investigation of TE insertion timing relative to the divergence of PB and BBs.

## Figures and Tables

**Table 1:** Brown bear fixed CAN SINE deletions overlapping UTRs (polar reference).

Gene Symbol	Scaffold (16 Total)	Deletion Start	Deletion End	Deletion Size	Gene Strand	TE Strand	Region	Phenotypes/Function
*BBS10*	NW_024424564.1	16586966	16587169	204	−	−	3′ UTR	body weight/obesity, leptin level, lipid, and energy metabolism
*CDH16*	NW_024424341.1	22228485	22228695	211	−	+	3′ UTR	B-cell number, IgG level, and kidney development
*FYB2*	NW_024423564.1	58585852	58586055	204	+	+	3′ UTR	snout morphology, body fat level
*MDH2*	NW_024424452.1	9101197	9101421	225	−	+	5′ UTR	decreased circulating glucose level, embryo size, brain development
*NEPRO*	NW_024426552.1	56680208	56680407	200	−	+	5′ UTR	kidney morphology
*PARVB*	NW_024424564.1	51878360	51878560	201	+	+	3′ UTR	cholesterol level, weight, B/T-cell number, bone mineral density
*PDSS2*	NW_024423675.1	18483400	18483600	201	−	−	3′ UTR	cholesterol level, weight, posture, kidney morphology
*PIGH*	NW_024425008.1	31674393	31674600	208	+	−	3′ UTR	circulating cholesterol
*RECQL4*	NW_024427107.1	80502573	80502778	206	−	−	3′ UTR	hair morphology, pigmentation, body size/fat
*RNF170*	NW_024425341.1	1742136	1742333	198	+	−	5′ UTR	innate immune responses, TLR3 signaling, increased thermal nociceptive threshold
*RNF213*	NW_024424897.1	2371468	2371669	202	−	−	3′ UTR	body weight, glucose level/decreased insulin secretion
*SERPINI1*	NW_024426552.1	93636368	93636569	202	−	+	5′ UTR	adipose tissue/craniofacial morphology
*SLC29A3*	NW_024426885.1	53992950	53993167	218	−	+	5′ UTR	bacterial infection, hair pigment/abnormal skin morphology, circulating cholesterol level
*SMARCA4*	NW_024423786.1	49476401	49476606	206	−	−	3′ UTR	enlarged heart, T cell differentiation, skin texture
*WDR35*	NW_024426774.1	14914534	14914743	210	−	+	3′ UTR	skeletal abnormalities, kidney morphology
*ZBTB37*	NW_024424452.1	77702270	77702469	200	+	−	5′ UTR	immune system regulation
*ZBTB8OS*	NW_024425786.1	24314281	24314484	204	−	−	3′ UTR	preweaning lethality, increased prepulse inhibition
*ZSCAN2*	NW_024425230.1	21888745	21888951	207	−	−	5′ UTR	zinc finger transcription factor
*ZSCAN9*	NW_024425564.1	8281260	8281461	202	−	+	5′ UTR	zinc finger transcription factor

Genomic coordinates are provided relative to the PB reference genome. Gene names in blue indicate a fixed insertion in the outgroup (see Section 3.8).

**Table 2:** Polar bear fixed CAN SINE deletions overlapping UTRs (brown reference).

Gene Symbol	Scaffold (5 Total)	Deletion Start	Deletion End	Size	Gene Strand	TE Strand	Region	Phenotype/Function
*CFLAR*	NW_026622763.1	76302344	76302565	222	+	−	5′ UTR	body size, B cell number, respiration and oxygen use
*EPN2*	NW_026622808.1	1961708	1961908	201	+	−	3′ UTR	lipid binding
*GPN2*	NW_026623008.1	19527285	19527479	195	−	+	3′ UTR	carbohydrate derivative binding
*ORC6*	NW_026622863.1	5605301	5605493	193	+	−	3′ UTR	growth, preweaning lethality
*RHBDD1*	NW_026622763.1	97756854	97757053	200	+	+	5′ UTR	weight loss susceptibility
*UACA*	NW_026622963.1	6494147	6494344	198	−	−	5′ UTR	cholesterol level
*USB1*	NW_026622863.1	14863442	14863652	211	+	+	3′ UTR	craniofacial, cardiovascular system, growth

Genomic coordinates are provided relative to the BB reference genome. Gene names in blue indicate a fixed insertion in the outgroup; gene names in red indicate a mixed ancestral state (see Section 3.8).

**Table 3:** Brown bear CAN SINE deletions upstream of 5′ UTR (polar reference).

Gene Symbol	Scaffold (16 Total)	Deletion Start	Deletion End	Deletion Size	Gene Strand	TE Strand	Distance to TSS	Phenotypes/Function
*CDH4*	NW_024424675.1	45772908	45773115	208	+	−	3046	kidney morphology, nervous system
*CHIC2*	NW_024426996.1	41038250	41038455	206	+	+	2338	abnormal coat, hair pigmentation, enlarged heart
*DLGAP1*	NW_024424230.1	51657616	51657830	215	−	−	4794	skin morphology, eye morphology
*EFHC2*	NW_024423319.1	86504293	86504502	210	+	+	4983	neuronal/behavioral traits
*GLE1*	NW_024424119.1	53100303	53100514	212	+	−	265	neurodevelopmental phenotypes; enlarged heart
*GMNC*	NW_024426552.1	73509199	73509399	201	+	+	2085	body weight
*GNL3*	NW_024423786.1	36782157	36782370	213	−	+	2425	brain development, spinal cord size
*IGDCC4*	NW_024425230.1	37589545	37589776	232	+	+	2085	body weight, viral susceptibility
*MEF2A*	NW_024425230.1	9375629	9375835	207	−	+	3057	cardiac and vascular development, dilated heart, right ventricle
*NLRP6*	NW_024424786.1	47373243	47373452	209	−	−	2467	immunity, body weight
*OCRL*	NW_024423319.1	3619820	3620027	208	+	+	3890	renal function
*RANBP3L*	NW_024423897.1	35807722	35807926	205	+	−	2633	craniofacial/skeletal traits
*RECK*	NW_024424119.1	25399122	25399328	207	+	−	3303	abnormal vascular remodeling/abnormal limb morphology
*RESF1*	NW_024425119.1	28585318	28585517	200	+	−	3693	cardiovascular, eye morphology, respiratory
*RGS22*	NW_024427107.1	44890418	44890624	207	−	−	4991	reproductive/sperm function
*SDHC*	NW_024424452.1	44926539	44926735	197	+	−	1932	weight loss, bone mineral density, glucose level
*SHC4*	NW_024426119.1	24317160	24317356	197	+	−	4361	weight loss, bone mineral density, grip strength
*SNTG2*	NW_024426774.1	9066901	9067110	210	−	+	4748	neuronal signaling;
*SPATA6*	NW_024423564.1	65763234	65763447	214	+	−	875	short tibia, pulmonary ventilation
*SYN2*	NW_024423786.1	6560919	6561114	196	−	−	3923	synaptic function

Genomic coordinates are provided relative to the PB reference genome. Gene names in blue indicate a fixed insertion in the outgroup (see Section 3.8).

**Table 4:** Polar bear fixed CAN SINE deletions upstream of 5′ UTR (brown reference).

Gene Symbol	Scaffold (10 Total)	Deletion Start	Deletion End	Deletion Size	Gene Strand	TE Strand	Distance to TSS	Phenotype/Function
*CDKL4*	NW_026623100.1	55048348	55048547	200	+	+	826	cataract
*CT83*	NC_079873.1	91104851	91105049	199	−	+	1199	cancer/testis antigen
*CUNH15orf39 ^a^*	NW_026622963.1	10280561	10280753	193	+	−	2695	inhibits inflammatory response
*GALNT18*	NW_026622908.1	2661079	2661279	201	−	+	1751	lean body mass, NK cell number, body fat amount, red blood cell count.
*GPI*	NW_026622863.1	40202337	40202541	205	+	−	3462	embryonic growth
*KLRD1*	NW_026622941.1	10078765	10078975	211	+	−	1065	signaling, lipid metabolism
*MARCHF4*	NW_026622763.1	89117065	89117262	198	−	−	4706	DNA/RNA binding, protein metabolism
*MMP24*	NW_026622830.1	23112592	23112801	210	−	−	4858	thermal pain nociception
*MTLN*	NW_026623100.1	51302603	51302798	196	+	−	1721	oxidative phosphorylation, fatty acid beta-oxidation, cardiac muscle tissue morphology
*SERTAD1*	NW_026622863.1	45001472	45001689	218	−	−	2380	insulin secretion
*SLC25A51*	NW_026622852.1	27834835	27835044	210	−	−	4596	carbohydrate metabolism
*SULT2B1*	NW_026622863.1	50085685	50085888	204	+	−	2703	cholesterol level
*TEX30*	NW_026622764.1	8684755	8684956	202	+	−	1952	embryo development
*TTC32*	NW_026623100.1	70743488	70743688	201	+	−	659	increased circulating bilirubin level

Genomic coordinates are provided relative to the BB reference genome. *^a^ CUNH15orf39* is a bear homolog of *C15orf39* (human)/*C8h15orf39* (rat) with no entry in MGI or GeneCards. The functional annotation is derived from the Rat Genome Database (RGD) [50] entry for *C8h15orf39*. Gene names in blue indicate a fixed insertion in the outgroup (see Section 3.8).

**Table 5:** Brown bear fixed CAN SINE deletions downstream of 3′ UTR (polar reference).

Gene Symbol	Scaffold (10 Total)	Deletion Start	Deletion End	Size	Gene Strand	TE Strand	Distance to TES	Phenotype/Function
*EPG5*	NW_024424230.1	27089216	27089412	197	+	−	1045	bone development, body immunity
*GLI2*	NW_024423343.1	99864611	99864822	212	+	+	874	bone morphology
*HAUS1*	NW_024424230.1	26876858	26877063	206	−	−	82	lipid metabolism, immune system
*IHO1*	NW_024423786.1	39661355	39661560	206	−	−	514	craniofacial, digestive system, cardiovascular
*MATN2*	NW_024427107.1	43165885	43166082	198	+	−	697	cellular organization
*OTOA*	NW_024424452.1	22096448	22096645	198	−	+	296	auditory system, hemoglobin levels
*PCDHB16*	NW_024426663.1	52921190	52921382	193	−	+	361	cell projection
*PGAM1*	NW_024426885.1	30193908	30194113	206	−	+	1082	metabolism, decreased oxygen, immunity
*RRAD*	NW_024424341.1	22241036	22241238	203	−	+	1031	heart rate, hemoglobin level
*SEMA4A*	NW_024424452.1	41150611	41150806	196	+	−	762	eye morphology
*SLTM*	NW_024426119.1	15584312	15584512	201	+	−	206	carbohydrate, protein, lipid binding
*TFR2*	NW_024424452.1	7495648	7495855	208	−	+	756	heart morphology, body weight, erythrocyte number
*TRNAD-GUC*	NW_024425564.1	9034215	9034420	206	−	−	788	tRNA
*VENTX*	NW_024426885.1	488367	488578	212	−	−	489	early development

Genomic coordinates are provided relative to the PB reference genome.

**Table 6:** Polar bear fixed CAN SINE deletions downstream of 3′ UTR (brown reference).

Gene Symbol	Scaffold (6 Total)	Deletion Start	Deletion End	Size	Gene Strand	TE Strand	Distance to TES	Phenotype/Function
*CLDN17*	NW_026623056.1	80630154	80630363	210	−	+	422	kidney morphology
*EIF3B*	NW_026622874.1	102669142	102669324	183	−	−	771	embryonic lethality
*FAM24B*	NW_026623089.1	8721951	8722157	207	+	−	69	hair pigmentation
*FBRS*	NW_026622874.1	88046408	88046615	208	+	−	827	cholesterol homeostasis
*MAP4K3*	NW_026623100.1	55048348	55048548	199	+	+	929	immunity
*TAF7*	NW_026623067.1	38421447	38421654	208	−	−	527	immunity
*ZNF706*	NW_026623078.1	52434067	52434292	226	−	+	468	hair pigmentation

Genomic coordinates are provided relative to the BB reference genome. Gene names in red indicate mixed ancestral state in the outgroup (see Section 3.8).

## Data Availability

Dataset files and custom code used are available at https://github.com/grigoriev-group/genome-comparison (accessed on 15 June 2026).

## Supplementary Materials

The following supporting information can be downloaded at https://www.mdpi.com/article/10.3390/cimb48060639/s1, https://github.com/grigoriev-group/genome-comparison (accessed on 15 June 2026).

## Abbreviations

The following abbreviations are used in this manuscript:

BB	Brown Bear
PB	Polar Bear
PBSD	Polar Bear-Specific Deletions
BBSD	Brown Bear-Specific Deletions
TEs	Transposable Elements
