# Supplementary material for: Recent Dominant Transposition Events Affect Gene Regulatory Regions, but Not Coding Sequences, in Polar and Brown Bear Genomes
Source: Curr Issues Mol Biol. 2026 Jun 20;48(6):639. doi: 10.3390/cimb48060639 (PMC13298077; doi:10.3390/cimb48060639)
Supplement: Supplementary file 1 [file cimb-48-00639-s001.zip › cimb-4360324-supplementary.pdf]

Supplementary Materials for Njagi et al. “Recent Dominant Transposition Events Affect Gene Regulatory Regions but Not Coding Sequences in Polar and Brown Bear Genomes”

Table S1. Sample Accession Numbers

Species	Sample ID	Accession Numbers
polar bear	BGI-polarbear-PB_36	SAMN02261805
polar bear	BGI-polarbear-PB_28	SAMN02261819
polar bear	BGI-polarbear-PB_72	SAMN02261840
polar bear	BGI-polarbear-PB_68	SAMN02261845
polar bear	BGI-polarbear-PB_62	SAMN02261851
polar bear	BGI-polarbear-PB_79	SAMN02261853
polar bear	BGI-polarbear-PB_47	SAMN02261854
polar bear	BGI-polarbear-PB_33	SAMN02261858
polar bear	BGI-polarbear-PB_12	SAMN02261865
polar bear	BGI-polarbear-PB_45	SAMN02261868
polar bear	BGI-polarbear-PB_42	SAMN02261870
polar bear	BGI-polarbear-PB_89	SAMN02261878
polar bear	BGI-polarbear-PB_105	SAMN02261880
brown bear	BGI-brownbear-20105434	SAMN02256313
brown bear	BGI-brownbear-20105373	SAMN02256314
brown bear	BGI-brownbear-7429	SAMN02256315
brown bear	BGI-brownbear-43157	SAMN02256316
brown bear	BGI-brownbear-56159	SAMN02256317
brown bear	BGI-brownbear-63855	SAMN02256318
brown bear	BGI-brownbear-63856	SAMN02256319
brown bear	BGI-brownbear-63863	SAMN02256320
brown bear	BGI-brownbear-63864	SAMN02256321
brown bear	BGI-brownbear-107596012	SAMN02256322
black bear	HA2	SAMN30214217
black bear	NVb84	SAMN46479199
black bear	NVb83	SAMN30214212
black bear	HA9	SAMN30214224
black bear	AK17117	SAMN30214203
black bear	HA5	SAMN30214220
black bear	NVg5	SAMN30214214
black bear	HA4	SAMN30214219
black bear	HA7	SAMN30214222
black bear	HA6	SAMN30214221
black bear	HA3	SAMN30214218

Summary of WGS samples used in this study, including species, sample identifiers, and NCBI SRA accession numbers. WGS data for polar and brown bears were originally generated by Liu et al. (2014), and black bear samples were generated by Clendenin et al. (2025).

Table S2: Reference Sequences Used

Species	Scientific Name	Assembly	RefSeq
polar bear	Ursus maritimus	ASM1731132v1	GCF_017311325.1
brown bear	Ursus arctos	UrsArc2.0	GCF_023065955.2
black bear	Ursus americanus	gsc_jax_bbear_1.0	GCF_020975775.1

Table S3: Per-locus target-site evidence. Homologous sequences (Figure 1b) were generated by Pindel; TSD sequences were computed as the breakpoint-anchored longest k-mer match between the left and right CAN-SINE flanks (Figure 1a).

Affected Gene	Homologous Sequence Length	Homologous Sequence	TSD Length	TSD Sequence	TSD Chance
**From Table 1**					
BBS10	13	TTGTGACATTGTT	13	TTGTGACATTGTT	0.0001
CDH16	16	AGGAAGCTCTGCTTGG	16	AGGAAGCTCTGCTTGG	0.0001
FYB2	15	AGAATGCTGAAAAGT	15	AGAATGCTGAAAAGT	0.0001
MDH2	16	ACAACTGAAAATCTTG	16	ACAACTGAAAATCTTG	0.0001
NEPRO	16	AAGATAACCTGGTATA	16	AAGATAACCTGGTATA	0.0001
PARVB	17	AAAATGTTACTCAGACA	12	GTTACTCAGACA	0.0001
PDSS2	11	AATTTTTCTTT	11	AATTTTTCTTT	0.0001
PIGH	17	CTGATATCAGGTCTTTT	17	CTGATATCAGGTCTTTT	0.0001
RECQL4	15	TGCCCCCAAACTTCT	14	GCCCCCAAACTTCT	0.0001
RNF170	14	TGGAGTGAGTTTTT	14	TGGAGTGAGTTTTT	0.0001
RNF213	17	TCATCAAGGACATTCTT	17	TCATCAAGGACATTCTT	0.0001
SERPINI1	15	AAGAAATCCCTGCTG	15	AAGAAATCCCTGCTG	0.0001
SLC29A3	14	TAAAACCTCAGCGG	14	TAAAACCTCAGCGG	0.0001
SMARCA4	16	AAGCACAGGGCTCCTT	16	AAGCACAGGGCTCCTT	0.0001
WDR35	17	TAAAGCATCCTCATTGG	17	TAAAGCATCCTCATTGG	0.0001
ZBTB37	18	GCTATTAGCCACCTTTTA	18	GCTATTAGCCACCTTTTA	0.0001
ZBTB8OS	8	GTTTTGTG	8	GTTTTGTG	0.0001
ZSCAN2	18	CAAAGATGGTTATTTTTA	18	CAAAGATGGTTATTTTTA	0.0001
ZSCAN9	17	AAAAAAACACCCTCCAG	17	AAAAAAACACCCTCCAG	0.0001
**From Table 2**					
CFLAR	16	CACCTTGGCTATATTT	16	CACCTTGGCTATATTT	0.0001
EPN2	17	TAAAGAATGTCTTTTTT	17	TAAAGAATGTCTTTTTT	0.0001
GPN2	15	GCAACGTAAACTTTT	15	GCAACGTAAACTTTT	0.0001
ORC6	18	AAAACTTGTCTACTCTAG	18	AAAACTTGTCTACTCTAG	0.0001
RHBDD1	16	CTTTCTTGCTCATTCT	16	CTTTCTTGCTCATTCT	0.0001
UACA	17	AAAACCAAGAGGAGTCA	17	AAAACCAAGAGGAGTCA	0.0001
USB1	3	TAA	0		1.0000
**From Table 3**					

Affected Gene	Homologous Sequence Length	Homologous Sequence	TSD Length	TSD Sequence	TSD Chance
CDH4	16	TCTCTAGGGCTCTTTA	16	TCTCTAGGGCTCTTTA	0.0001
CHIC2	13	AAGATAAAGGGTG	13	AAGATAAAGGGTG	0.0001
DLGAP1	8	TGATTTCT	8	TGATTTCT	0.0001
EFHC2	10	AGAAAAACCA	10	AGAAAAACCA	0.0001
GLE1	17	CGAGCTGCCTCATTTTA	17	CGAGCTGCCTCATTTTA	0.0001
GMNC	18	AAAAAGAATGAAACCTTG	18	AAAAAGAATGAAACCTTG	0.0001
GNL3			17	AAAAACGTGTCCTTTGG	0.0001
IGDCC4	15	AGAAAGTTGACTAGT	15	AGAAAGTTGACTAGT	0.0001
MEF2A	15	AGAGAGAAACCACAG	15	AGAGAGAAACCACAG	0.0001
NLRP6			15	GCTCTGGTGTGTTCT	0.0001
OCRL	17	AGAATTTCATTTATTTG	17	AGAATTTCATTTATTTG	0.0001
RANBP3L	12	AGAGTGGATCTT	12	AGAGTGGATCTT	0.0001
RECK	17	CTGCCTTAGACATTTTA	17	CTGCCTTAGACATTTTA	0.0001
RESF1	19	TCATGTCAATTTGATTCTT	19	TCATGTCAATTTGATTCTT	0.0001
RGS22	15	TGGTTTTAATTTTTA	15	TGGTTTTAATTTTTA	0.0001
SDHC	17	CCCTCTTGCTTATTTTT	17	CCCTCTTGCTTATTTTT	0.0001
SHC4	12	CCTTGTGATTTT	12	CCTTGTGATTTT	0.0001
SNTG2	16	AAAATTAGCCCCCTAG	16	AAAATTAGCCCCCTAG	0.0001
SPATA6	9	AGTAATGTT	9	AGTAATGTT	0.0001
SYN2	18	CTATCCAGGTTTCTTTTT	18	CTATCCAGGTTTCTTTTT	0.0001
**From Table 4**					
CDKL4	16	CCACCTAGTAACTTTT	16	CCACCTAGTAACTTTT	0.0001
CT83	16	TCAACAAGTATTTCTT	16	TCAACAAGTATTTCTT	0.0001
CUNH15orf39	17	TAAGTGAGCTGTCTATT	17	TAAGTGAGCTGTCTATT	0.0001
GALNT18	18	CTCATTTTTCACATTCTT	18	CTCATTTTTCACATTCTT	0.0001
GPI	17	CTATTTTCAGATTTCTT	17	CTATTTTCAGATTTCTT	0.0001
KLRD1	19	TAAAAAAGAGTAGAATTTC	19	TAAAAAAGAGTAGAATTTC	0.0001
MARCHF4	18	AAGATATGTAGAATTCAG	18	AAGATATGTAGAATTCAG	0.0001
MMP24	13	AGAAGAGTGGAGT	13	AGAAGAGTGGAGT	0.0001
MTLN	15	GAGGGTTTTCTTCTT	15	GAGGGTTTTCTTCTT	0.0001
SERTAD1	3	AAA	3	AAA	0.0250
SLC25A51	16	AGAAGTGCAGTTGAGC	15	GAAGTGCAGTTGAGC	0.0001
SULT2B1	16	TAAAATATCATCATTT	16	TAAAATATCATCATTT	0.0001
TEX30	17	AGAAAACCCAGAAACGG	15	GAAAACCCAGAAACG	0.0001
TTC32	13	TATGAATAGAACT	13	TATGAATAGAACT	0.0001
**From Table 5**					
EPG5	14	TCTACTGGGTTTTT	14	TCTACTGGGTTTTT	0.0001
GLI2	17	AGAAAACCCATAGCCAG	17	AGAAAACCCATAGCCAG	0.0001

Affected Gene	Homologous Sequence Length	Homologous Sequence	TSD Length	TSD Sequence	TSD Chance
HAUS1	14	CTCCTTTTACTTTG	14	CTCCTTTTACTTTG	0.0001
IHO1	15	CCATTTGGTTTTCTT	15	CCATTTGGTTTTCTT	0.0001
MATN2	17	TAACCAGCCTCTTCTTT	17	TAACCAGCCTCTTCTTT	0.0001
OTOA	15	AGAAGGGAAGGGGAG	15	AGAAGGGAAGGGGAG	0.0001
PCDHB16	0		17	AAAAACTTAACAGGGAG	0.0001
PGAM1	10	AAGAGTTCTA	10	AAGAGTTCTA	0.0001
RRAD	11	AAGACAGGTCC	11	AAGACAGGTCC	0.0001
SEMA4A	18	TAAAAAATGTTTTTATTT	18	TAAAAAATGTTTTTATTT	0.0001
SLTM	17	AGCTTTTTTTTTTTTTT	14	TTTTTTTTTTTTTT	0.0001
TFR2	15	AAGAGTCTGACATCT	15	AAGAGTCTGACATCT	0.0001
TRNAD-GUC	16	CATACTCATTTTTCTT	16	CATACTCATTTTTCTT	0.0001
VENTX	14	TAAGATTTTATTTT	14	TAAGATTTTATTTT	0.0001
**From Table 6**					
CLDN17	18	AAGAAATAATTTGGAGGA	18	AAGAAATAATTTGGAGGA	0.0001
EIF3B	16	CCTGGACCTCTTCCTT	16	CCTGGACCTCTTCCTT	0.0001
FAM24B	15	ATAAATGCACTTACA	15	ATAAATGCACTTACA	0.0001
FBRS	18	AGAAATCGGAGACGGTTG	18	AGAAATCGGAGACGGTTG	0.0001
MAP4K3	16	CCACCTAGTAACTTTT	15	CACCTAGTAACTTTT	0.0001
TAF7	11	GAAAATTGTTA	11	GAAAATTGTTA	0.0001
ZNF706	18	TAAAACAGAAGCTATATT	18	TAAAACAGAAGCTATATT	0.0001

Note: TSD lengths are the exact break-point-anchored longest k-mer match between left and right flanks. USB1 = 0 and SERTAD1 = 3 because the duplicated target site carries a single A/T point difference between the two copies (USB1: TAA[T/A]CCTTGGTGTCAG, one discordant position; SERTAD1: [T/A]AATAA[T/A]), which breaks the exact match, although a longer TSD is evident. Only one frequent event was observed (USB1- yellow highlight).

Table S4: Complete reciprocal validation summary for genes in tables 1-6 in the main text

Table 1*							
Gene	DEL Region	INS Region	INS Scaffold	Repeat Start	Repeat End	Distance	Case
BBS10	3' UTR	downstream 3' UTR	NW_026622886.1	34170570	34170582	1119	1
CDH16	3' UTR	downstream 3' UTR	NW_026622863.1	22116370	22116385	1080	1
FYB2	3' UTR	overlap	NW_026622786.1	60193810	60193824		
MDH2	5' UTR	downstream 5' UTR	NW_026622874.1	95546499	95546514	2706	4
NEPRO	5' UTR	upstream 5' UTR	NW_026623056.1	39071484	39071499	1348	3
PARVB	3' UTR	not found by BLAST					
PDSS2	3' UTR	upstream 3' UTR	NW_026622797.1	51760066	51760076	37506	2
PIGH	3' UTR	overlap	NW_026622930.1	32370487	32370503		
RECQL4	3' UTR	overlap	NW_026623078.1	86388716	86388729		
RNF170	5' UTR	upstream 5' UTR; downstream 5' UTR	NW_026622952.1	1841837	1841850	3056;894	7
RNF213	3' UTR	upstream 3' UTR	NW_026622919.1	2349890	2349906	22781	2
SERPINI1	5' UTR	downstream 5' UTR	NW_026623056.1	2423090	2423104	33707	4

SLC29A3	5' UTR	downstream 5' UTR	NW_026623089.1	53241799	53241812	12309	4
SMARCA4	3' UTR	downstream 3' UTR	NW_026622808.1	19669633	19669648	5345	1
WDR35	3' UTR	overlap	NW_026623100.1	70738977	70738993		
ZBTB37	5' UTR	downstream 5' UTR	NW_026622874.1	14730695	14730712	1368	4
ZBTB8OS	3' UTR	overlap	NW_026623008.1	23907797	23907804		
ZSCAN2	5' UTR	between 2 sections of 5' UTR	NW_026622963.1	34289250	34289267	1239	5
ZSCAN9	5' UTR	overlap	NW_026622997.1	1325218	1325234		

Table 2*

Gene	DEL Region	INS Region	INS Scaffold	Repeat Start	Repeat End	Distance	Notes
CFLAR	5' UTR	downstream 5' UTR	NW_024423343.1	33564885	33564900	276	4
EPN2	3' UTR	downstream 3' UTR	NW_024423786.1	68459329	68459345	1748	1
GPN2	3' UTR	overlap	NW_024425786.1	19911478	19911492		
ORC6	3' UTR	downstream 3' UTR	NW_024424341.1	5604765	5604782	49	1
RHBDD1	5' UTR	upstream 5' UTR; downstream 5' UTR	NW_024423343.1	11944342	11944357	8087;4703	7
UACA	5' UTR	upstream 5' UTR; downstream 5' UTR	NW_024425230.1	33013816	33013832	6164;42043	7
USB1	3' UTR	downstream 3' UTR	NW_024424341.1	14920008	14920023	712	1

Table 3

Gene	DEL Dist upstream	INS Region	Scaffold	Repeat Start	Repeat End	Distance	Notes
CDH4	3046	upstream 5' UTR	NW_026622830.1	2424295	2424310	3018	
CHIC2	2338	upstream 5' UTR	NW_026623111.1	40992112	40992124	2215	
DLGAP1	4794	upstream 5' UTR	NW_026622841.1	51229397	51229404	10278	6
EFHC2	4983	upstream 5' UTR	NC_079873.1	37242197	37242206	4958	
GLE1	265	upstream 5' UTR	NW_026622852.1	53719612	53719628	237	
GMNC	2085	upstream 5' UTR	NW_026623056.1	22396013	22396030	177627	6
IGDCC4	2085	downstream 5' UTR	NW_026622963.1	1969309	1969323	4536	
MEF2A	3057	upstream 5' UTR	NW_026622963.1	21902480	21902494	2998	
OCRL	3890	upstream 5' UTR	NC_079873.1	1.02E+08	1.02E+08	5592	
RANBP3L	2633	upstream 5' UTR	NW_026622819.1	27634503	27634514	2420	
RECK	3303	upstream 5' UTR	NW_026622852.1	26255061	26255077	3246	
RESF1	3693	upstream 5' UTR	NW_026622941.1	28542661	28542679	3681	
RGS22	4991	upstream 5' UTR	NW_026623078.1	51519121	51519135	4766	
SDHC	1932	upstream 5' UTR	NW_026622874.1	60356631	60356647	1626	
SHC4	4361	upstream 5' UTR	NW_026623050.1	14861112	14861123	4404	
SNTG2	4748	upstream 5' UTR; downstream 5' UTR	NW_026623100.1	84838241	84838256	29513;18767	7
SPATA6	875	upstream 5' UTR	NW_026622786.1	67294405	67294413	842	
SYN2	3923	upstream 5' UTR	NW_026622808.1	65482210	65482227	3044	

Table 4

Gene	DEL Dist upstream	INS Region	Scaffold	Repeat Start	Repeat End	Distance	Notes

CDKL4	826	upstream 5' UTR	NW_024426774.1	30812291	30812306	14616	6
CT83	1199	upstream 5' UTR	NW_024423319.1	32940052	32940067	1181	
CUNH15orf39	2695	upstream 5' UTR	NW_024425230.1	29125569	29125585	2667	
GALNT18	1751	upstream 5' UTR	NW_024424786.1	976906	976923	1759	
GPI	3462	upstream 5' UTR	NW_024424341.1	40481441	40481457	3504	
KLRD1	1065	upstream 5' UTR	NW_024425119.1	10237602	10237620	1095	
MARCHF4	4706	upstream 5' UTR	NW_024423343.1	20636635	20636652	4680	
MMP24	4858	upstream 5' UTR	NW_024424675.1	24976745	24976757	22219	6
MTLN	1721	upstream 5' UTR	NW_024426774.1	34580972	34580987	3870	
SERTAD1	2380	upstream 5' UTR	NW_024424341.1	51344732	51344734	2394	
SLC25A51	4596	upstream 5' UTR	NW_024424119.1	26998239	26998253	4644	
SULT2B1	2703	upstream 5' UTR	NW_024424341.1	56565939	56565954	1792	
TEX30	1952	upstream 5' UTR	NW_024423344.1	70816834	70816848	1940	
TTC32	659	upstream 5' UTR	NW_024426774.1	14910035	14910047	640	

Table 5

Affected Gene	DEL Dist downstream	INS Region	Scaffold	Repeat Start	Repeat End	Distance	Notes
EPG5	1045	downstream 3' UTR	NW_026622841.1	26836436	26836449	1029	
GLI2	874	downstream 3' UTR	NW_026622763.1	10686923	10686939	995	
HAUS1	82	downstream 3' UTR	NW_026622841.1	26624689	26624702	68	
IHO1	514	downstream 3' UTR	NW_026622808.1	32611174	32611188	501	
MATN2	697	downstream 3' UTR	NW_026623078.1	49794997	49795013	679	
OTOA	296	downstream 3' UTR	NW_026622874.1	82595715	82595729	1253	1
PGAM1	1082	downstream 3' UTR	NW_026623089.1	29686020	29686029	1072	
RRAD	1031	downstream 3' UTR	NW_026622863.1	22128717	22128727	1020	
SEMA4A	762	downstream 3' UTR	NW_026622874.1	63991485	63991502	750	
SLTM	206	downstream 3' UTR	NW_026623050.1	23406145	23406157	187	
TFR2	756	downstream 3' UTR	NW_026622874.1	97128099	97128113	737	
TRNAD-GUC	788	downstream 3' UTR	NW_026622997.1	2143616	2143631	772	
VENTX	489	downstream 3' UTR	NW_026623089.1	208320	208333	475	

Table 6

Affected Gene	DEL Dist downstream	INS Region	Scaffold	Repeat Start	Repeat End	Distance	Notes
CLDN17	422	downstream 3' UTR	NW_024426552.1	14444051	14444068	404	
EIF3B	771	downstream 3' UTR	NW_024424452.1	1861803	1861818	761	
FAM24B	69	downstream 3' UTR	NW_024426885.1	9065662	9065676	38	
FBRS	827	downstream 3' UTR	NW_024424452.1	16672796	16672813	803	
MAP4K3	929	downstream 3' UTR	NW_024426774.1	30812291	30812305	911	
TAF7	527	downstream 3' UTR	NW_024426663.1	52840786	52840796	517	
ZNF706	468	downstream 3' UTR	NW_024427107.1	45826297	45826314	2371	1

**In Tables 1 and 2, a distance value is not given if the reciprocal insertion position overlaps an exon.*

Figure S1. Genome Navigator visualization of a fixed deletion

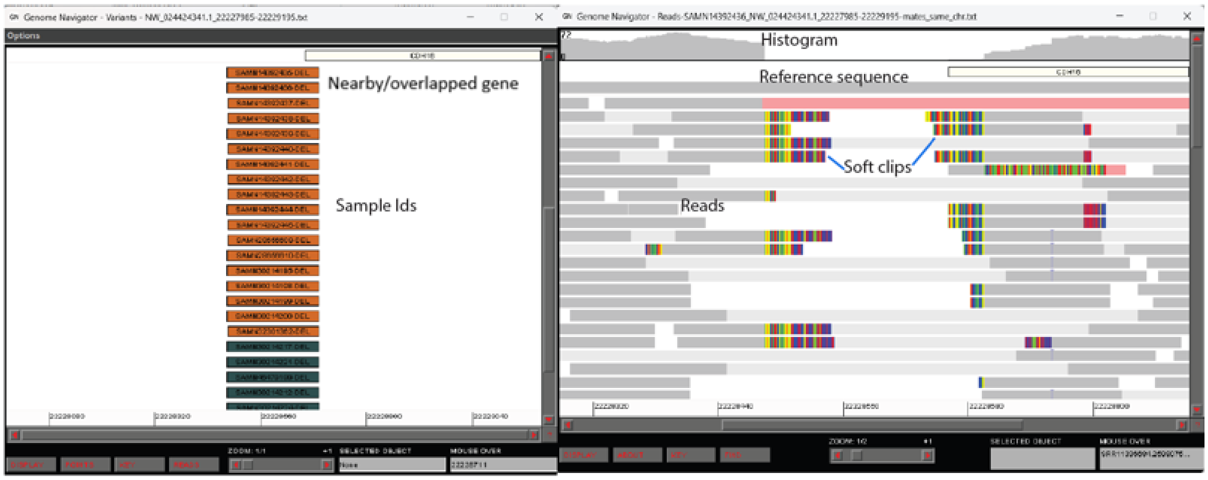

Genome Navigator visualization of a fixed deletion in brown bear samples aligned to a polar bear reference. A. *Variants view displaying a deletion on scaffold NW_024424341.1 overlapping the CDH16 gene. Each row represents an individual brown bear sample, with colored bars indicating deletion calls at concordant genomic positions across all samples, consistent with a fixed (homozygous) event. Sample identifiers are indicated inside the bars; genomic coordinates are shown along the bottom axis.* ***B.*** *Reads view for a single representative sample at the same locus. From top to bottom: the coverage histogram shows a sharp drop to zero across the deleted region; the reference sequence track displays the underlying nucleotide composition; soft-clipped bases (colored segments at read termini) mark the precise breakpoint positions flanking the deletion; and individual aligned reads (gray bars) span the junction, with gaps corresponding to the deleted CAN SINE element. The clustering of soft clips at both breakpoints provides independent support for the deletion boundaries.*

Figure S2. Positional Discrepancy Cases

Case 1. Insertion downstream of 3' UTR exon in polar samples brown reference

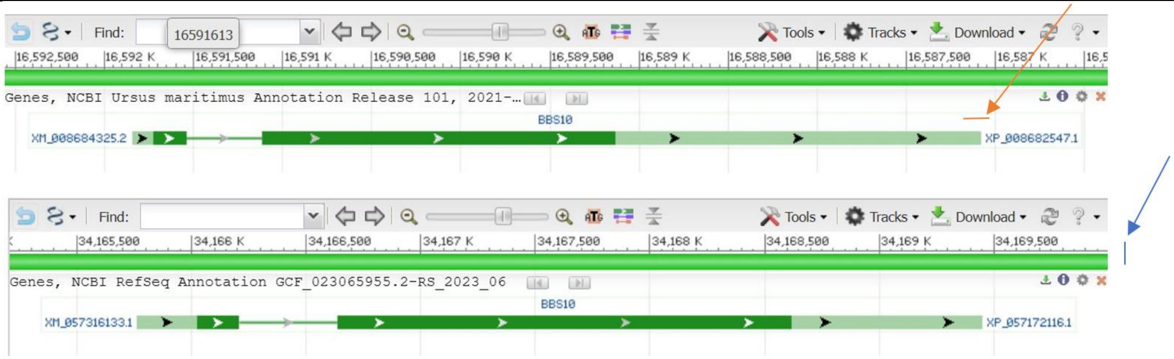

Polar reference (top). *Position of deletion observed in brown samples indicated by orange line and arrow. 3' UTR in polar is ~2000 bp long. In brown it is ~800 bp long.* ***Brown reference (bottom).*** *Blue arrow indicates position of insertion observed in polar samples. SV positions are about the same distance from the Stop codon.*

Case 2. Insertion in intron upstream of 3' UTR in polar samples brown reference. This usually occurs when multiple isoforms are present in one or both genomes.

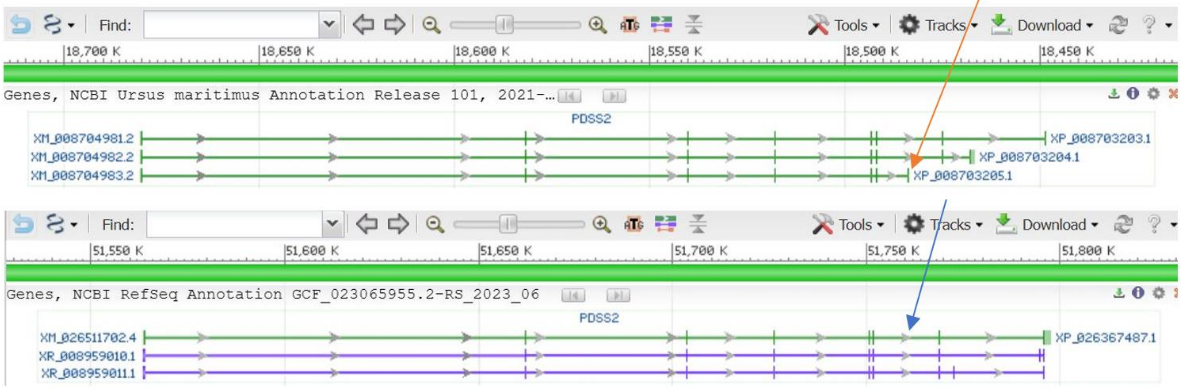

Polar reference (top).** Orange arrow indicates the 3' UTR that is overlapping the deletion in brown samples.* ***Brown reference (bottom) *has only 1 coding isoform. There is no 3' UTR at the corresponding position. Arrow indicates position of insertion observed in polar sample*

Case 3. Insertion upstream of 5' UTR exon in polar samples brown reference

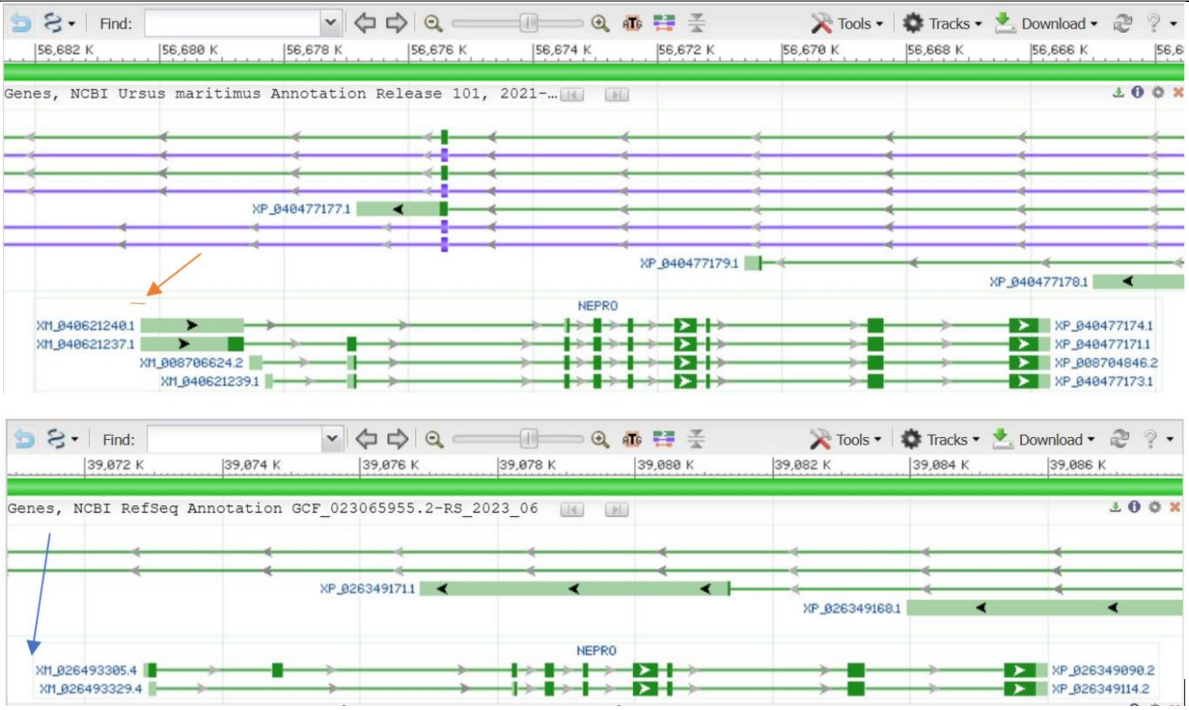

Top**. Polar reference and* ***Bottom. *Brown reference. The gene NEPRO is overlapped by another gene making the displays confusing. NEPRO is at the bottom of each screen capture. Orange arrow and line indicate position of deletion in brown samples polar reference. Blue arrow indicates position of insertion in polar samples brown reference. No analogous 5' UTR exists in brown reference to the 5' UTR overlapped in polar reference.*

Case 4. Insertion in intron downstream of 5’ UTR in polar samples brown reference

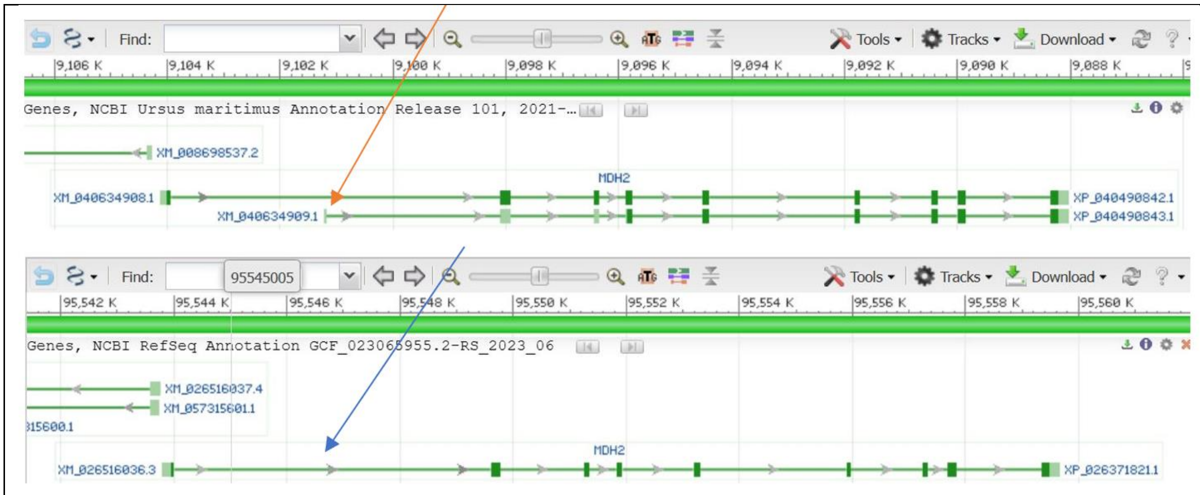

__Top__. Orange arrow and line indicate position of deletion in brown samples polar reference. ***Bottom.*** *Blue arrow indicates position of insertion in polar samples using brown reference. No analogous 5’ UTR exists in brown reference to the 5’ UTR overlapped in polar reference.*

Case 5. 5’ UTR is discontinuous. Insertion occurs in intron between portions of 5’ UTR.

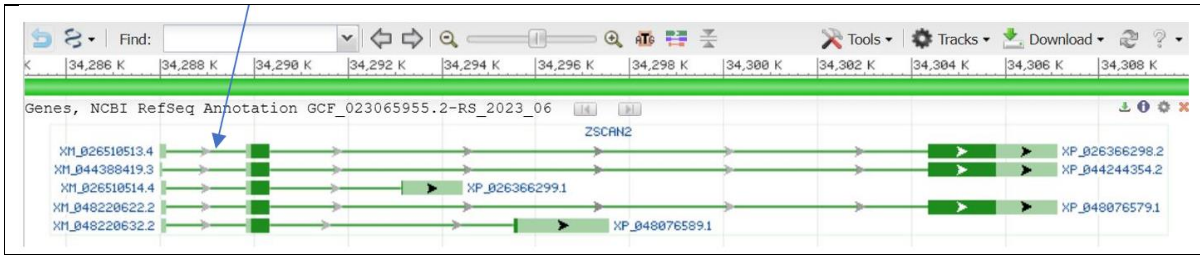

Case 6. Genes differ dramatically in size.

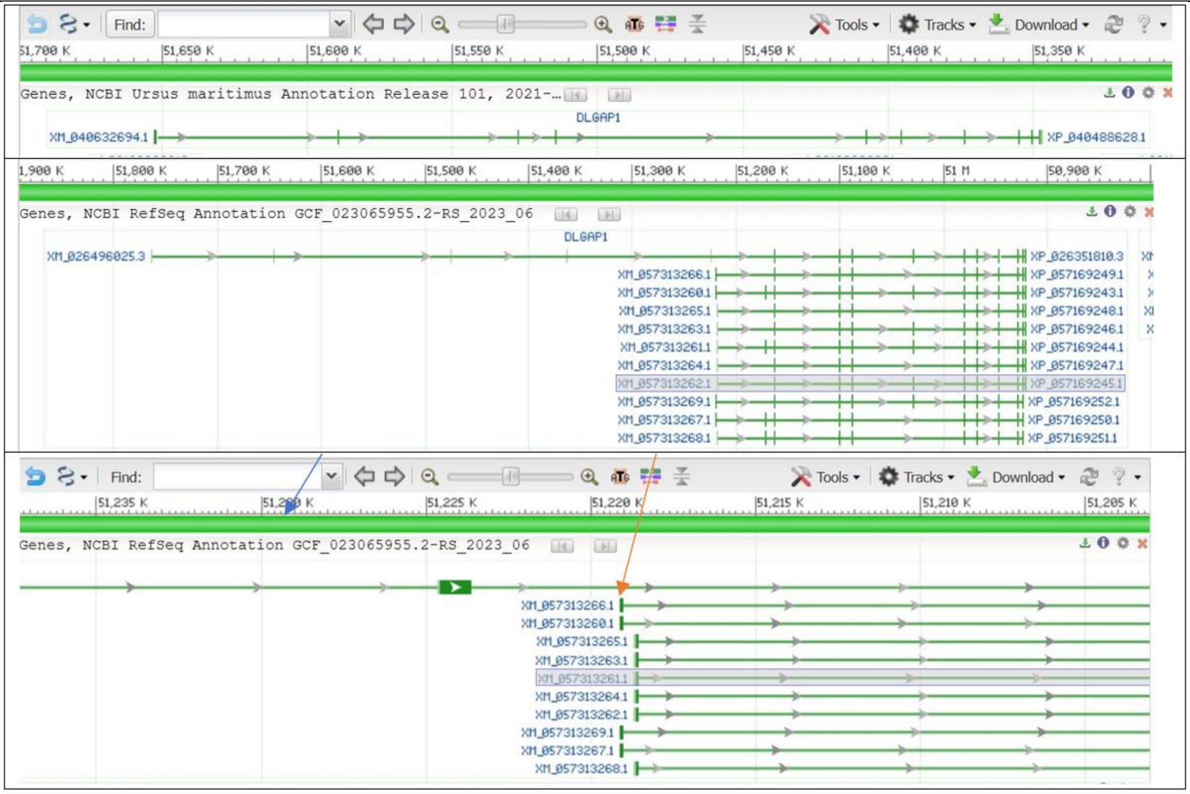

DLGAP1 (DLG Associated Protein 1):(Top. *Polar reference Gene length ~ 300000,* ***Middle.*** *Brown reference. Gene length ~800000* ***Bottom.*** *Brown ref zoom). The 215 bp deletion upstream of DLGAP1 (NW_024424230.1: 51,657,615-51,657,830) is fixed in all brown bear samples and located 4,794 bp from the nearest gene feature in the polar bear reference. We identified a corresponding insertion in the brown bear reference at NW_026622841.1: 51,229,397-51,229,404, but at a distance of 10,278 bp from the 5' UTR—more than twice the expected distance based on the polar reference coordinates. This discrepancy reflects substantial differences in gene structure between references: the polar bear DLGAP1 spans approximately 300 Kb, while the brown bear reference contains isoforms ranging from ~30 Kb to ~800 Kb. When comparing isoforms of similar length (XM_057313266.1 in polar bear and XM_040632694.1 in brown bear), the relative positions show better concordance. This case illustrates how reference-specific gene model differences can complicate positional comparisons of structural variants. Overlap is not conserved when either isoforms are different between the 2 genomes or the sizes of gene features are different*

Case 7. Insertion upstream 5' UTR and downstream 5' UTR

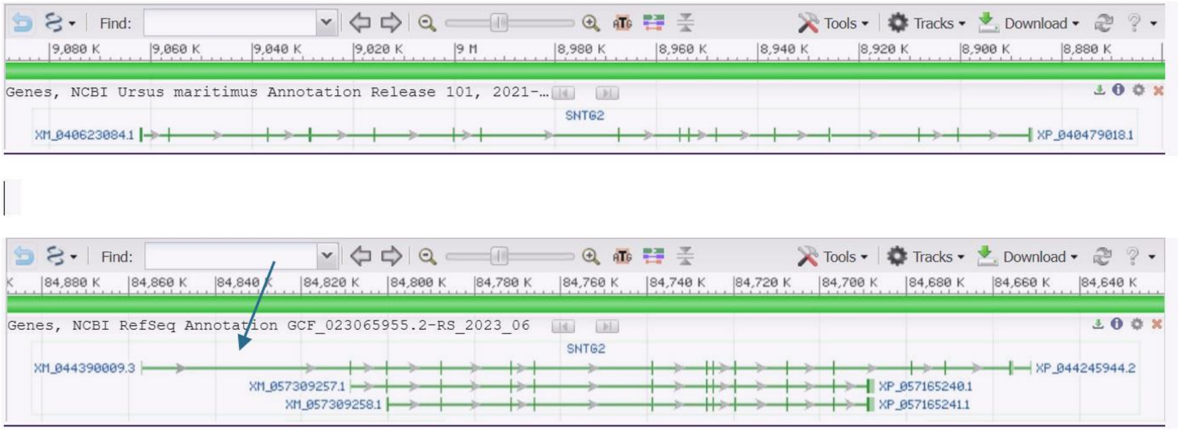

SNTG2. *Insertion is downstream of 5’ UTR in 1 isoforms and upstream of another.*

Figure S3: Two orientations for a CAN SINE Insertion

A

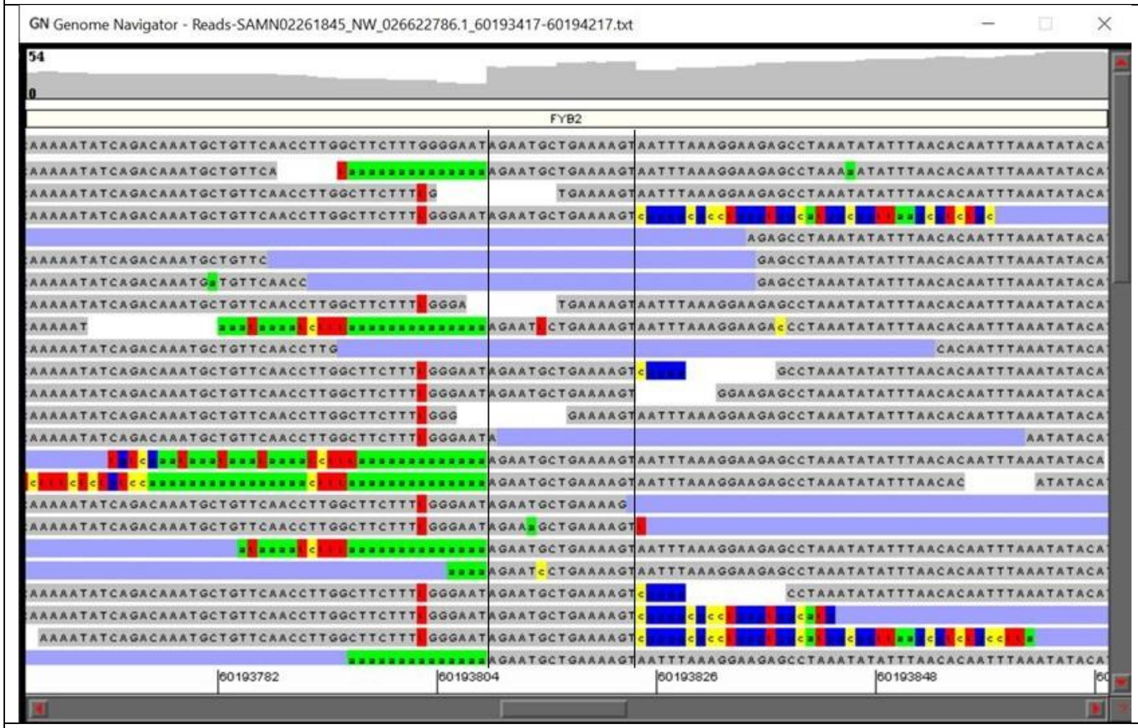

B

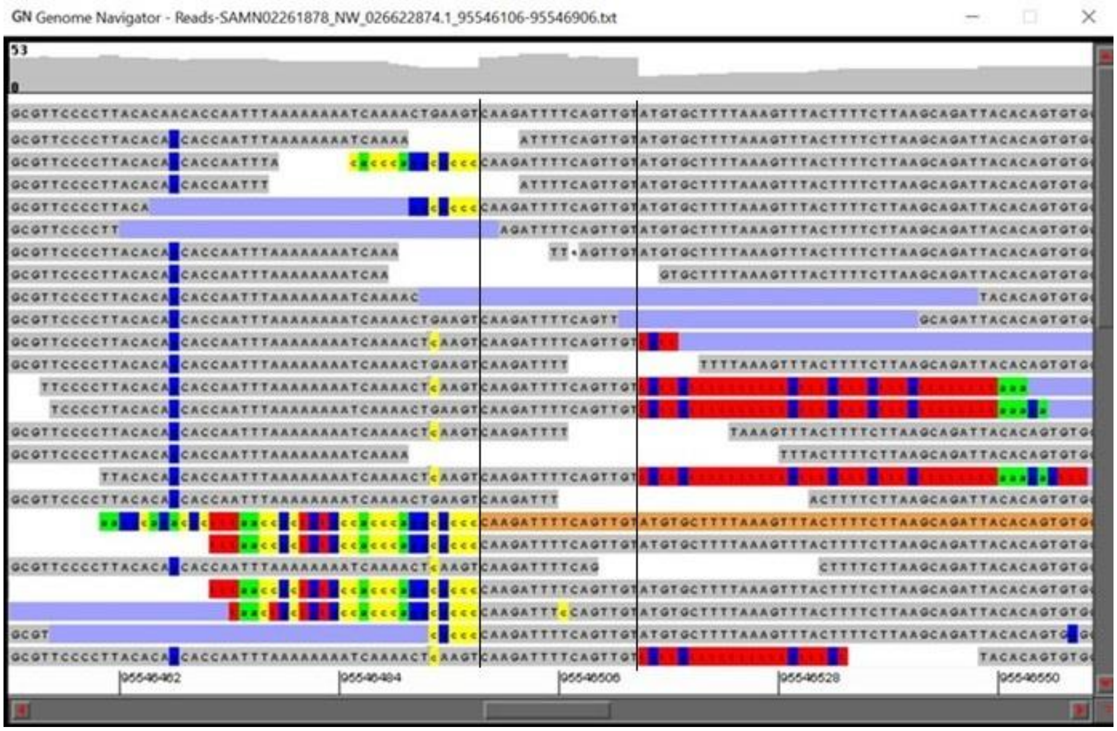

***Two orientations for a CAN SINE insertion**: **A**. This is the case that is illustrated in Fig 1b. CAN SINE on the plus strand, with poly A tail on the left. **B.** This is the reciprocal case when the CAN SINE is on the minus strand with T's on the right. The thin vertical lines show the insertion breakpoints in both cases.*

Figure S4: Visualization of heterozygous calls in reciprocal alignments using GN

A

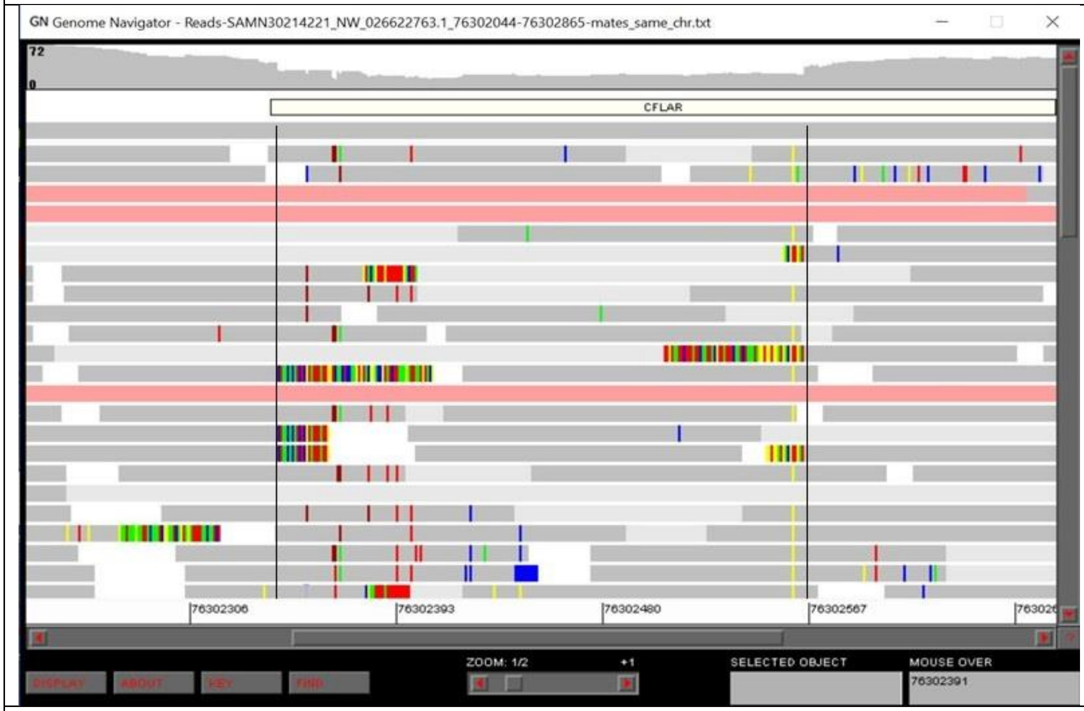

B

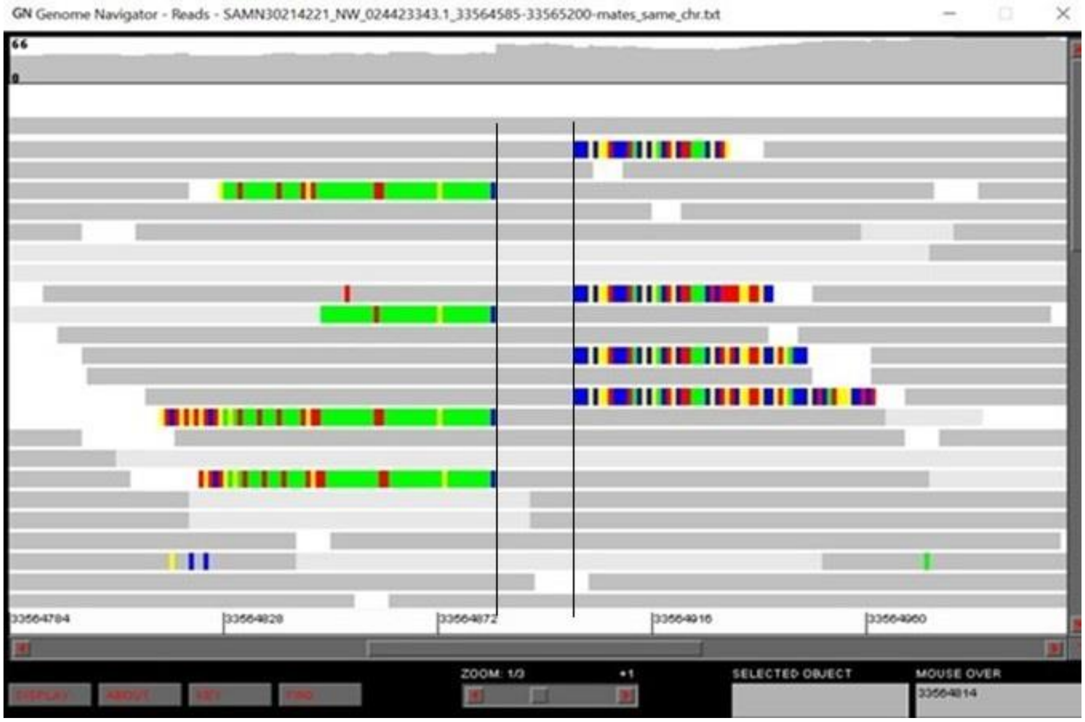

A. *GN view of heterozygous CAN SINE deletion affecting the gene CFLAR in a black bear sample aligned to the brown bear reference.* ***B****. GN view of a heterozygous insertion in the same sample aligned to polar bear reference. Notice the amplification is much lower than in the homozygous insertions in previous figures.*
